# Classical formula Taohe Chengqi decoction as an adjuvant therapy for sepsis - a systematic review and meta-analysis of randomized controlled trials

**DOI:** 10.3389/fphar.2025.1499280

**Published:** 2025-09-02

**Authors:** Youzhu Su, Rui Su, Chen Shen, Xinxin Liu, Xiao Xiao, Xuefei Wang, Zhijun Bu, Lingyao Kong, Jianping Liu

**Affiliations:** ^1^ Centre for Evidence-Based Chinese Medicine, Beijing University of Chinese Medicine, Beijing, China; ^2^ Beijing Hospital of Traditional Chinese Medicine, Capital Medical University, Beijing, China

**Keywords:** systematic review, Taohe Chengqi, Chinese herbal medicine, sepsis, randomized controlled trials

## Abstract

**Objectives:**

This systematic review aimed to evaluate the clinical efficacy and safety of Taohe Chengqi (THCQ) decoction as an adjuvant therapy for sepsis, and to provide evidence for clinical practice.

**Methods:**

Eight databases were systematically searched from inception until June 2024. The study included randomized controlled trials (RCTs) involving sepsis patients, where THCQ was used as an adjunctive therapy alongside conventional treatments. The primary outcome assessed was 28-day mortality rates, secondary outcomes included severity scores, inflammatory and coagulation markers, and adverse events. Two authors independently conducted the literature screening, data extraction, and evaluation of methodological quality. Meta-analysis was performed using RevMan 5.4.1 and Stata 17 software. The Risk of Bias (ROB) 2.0 and GRADE were employed for quality assessment.

**Results:**

A total of sixteen RCTs involving 1,034 participants were included. Most of the studies were rated as having “some concerns” according to the ROB 2.0. Compared with conventional treatments, THCQ plus conventional treatments resulted in lower 28-day mortality rate (RR = 0.57, 95% CI [0.38, 0.86]; low-certainty), lower Acute Physiology and Chronic Health Evaluation II score (APACHE-Ⅱ) (MD = −2.37, 95% CI [-3.12, −1.63]; low-certainty), lower Sequential Organ Failure Assessment score (SOFA) (MD = −1.41, 95% CI [-2.12, −0.70]; low-certainty), lower white blood cell (WBC) (MD = −1.78 109/L, 95% CI [-2.97, −0.59]; low-certainty), lower procalcitonin (PCT) (MD = −1.20 ng/mL, 95% CI [-1.71, −0.69]; low-certainty), lower C-reactive protein (CRP) (MD = −9.82 mg/L, 95% CI [-13.98, −5.66]; low-certainty), and no serious adverse effects were observed (RR = 0.67, 95% CI [0.12, 3.71]; very low-certainty).

**Conclusion:**

Chinese medicine formula THCQ may offer potential benefits as an adjunctive treatment for sepsis patients, suggesting a reduction in the 28-day mortality rate, improvements in inflammatory markers, and enhancement of coagulation function, with no severe adverse reactions observed. Given the low quality of the included studies, the findings should be interpreted with caution. Future large-scale, multicenter RCTs are needed to confirm these findings and provide robust evidence.

**Systematic Review Registration:**

https://www.crd.york.ac.uk/PROSPERO/view/CRD42024562595, identifier CRD42024562595.

## 1 Introduction

Sepsis is a life-threatening condition characterized by a systemic inflammatory response to infection and is commonly seen in patients with severe trauma or infectious diseases (Singer M. et al., 2016). Globally, there are over 19 million cases of sepsis each year, with approximately six million deaths (Prescott and Angus, 2018). The mortality rate is even higher in intensive care units (Dombrovskiy V. Y. et al., 2007). In high-income countries, the average hospital cost for a sepsis patient exceeds $32,000 per case (Arefian et al., 2017). Current clinical management of sepsis relies on antibiotics and supportive care (such as fluid resuscitation and vasopressors) (Rhodes A. et al., 2017). However, these measures have limited effect, and the mortality rate for sepsis remains high (Reinhart K. et al., 2017). In addition, such measures may lead to antibiotic resistance and poor recovery of organ function (van Donge T. et al., 2018; Gudiol C. et al., 2021). Under these circumstances, some people resort to complementary and alternative medicine.

In traditional Chinese medicine (TCM), there is no direct equivalent to “sepsis” in classical terminology. However, based on its clinical manifestations (high fever, systemic inflammation, and multi-organ dysfunction), sepsis is often categorized under the domain of “external contraction febrile disease” (Li, 2015). According to TCM theory, the pathogenesis of sepsis is generally understood as a result of zheng qi deficiency and stasis obstructing the collaterals, which leads to the accumulation of interior heat toxin, blood stasis, and phlegm dampness, eventually impairing the function of vital organs (Zhao H. F. et al., 2017). Based on this theoretical framework, classical TCM treatment strategies emphasize “facilitating bowel movements” and “promoting blood circulation to resolve stasis” as key principles for managing sepsis-like conditions (Wang, 2018). Taohe Chengqi decoction (THCQ), a well-known formula first recorded in the Treatise on Febrile Diseases (Shang Han Lun) by Zhang Zhongjing during the Han Dynasty (2223 years ago), was historically prescribed for “blood stasis with heat accumulation in the lower jiao” (Zhang Z. Z. Han Dynasty). Recognized in the first “Catalogue of Ancient Classical TCM Formulas” (https://www.gov.cn/zhengce/zhengceku/2018-12/31/content_5429153.htm), THCQ comprises *Prunus persica* (L.) Batsch [Rosaceae; *Persicae Semen*] (Tao Ren), *Rheum palmatum* L. [Polygonaceae; *Rhei Radix et Rhizoma*] (Da Huang), *Cinnamomum cassia* (L.) J. Presl [Lauraceae; *Cinnamomi ramulus*] (Gui Zhi), *Glycyrrhiza uralensis* Fisch. ex DC. [Fabaceae; *Glycyrrhizae Radix et Rhizoma*] (Gan Cao), and *Natrii Sulfas* [Sodium sulfate] (Mang Xiao), basic information of drug composition in THCQ was shown in Supplementary Appendix S1; Table1. Some preclinical studies have provided evidence for the use of THCQ in sepsis. The active metabolites in THCQ can protect myocardial cells by activating the Nrf2 signaling pathway, which has the potential to reduce inflammation and regulate the immune response, thereby reducing the mortality of sepsis mice (Deng et al., 2024; Lu S. M. et al., 2024). Therefore, we aim to explore the efficacy and safety of using THCQ in combination with conventional medical treatment for sepsis patients through a systematic review and meta-analysis.

**TABLE 1:** Basic characteristics of the included studies.

Study ID	Country	Sample size (I/C)	Gender (male/female)		Age (mean years)		Treatment Duration	Intervention	Control	Outcomes
			(I)	(C)	(I)	(C)				
Bao (2013)	China	29/31	17/12	21/10	76.14 ± 11.3	75.26 ± 11.29	7d	THCQ + C	FR, AT, VP, IA, GC, OST	4, 10, 11
Chen et al. (2021)	China	60/60	33/27	34/26	46.9 ± 6.2	47.3 ± 6.5	7d	THCQ + C	FR, AT, VP, GC, NS, OST	2, 3, 5, 6, 7
Jin et al. (2020)	China	34/34	24/10	20/14	75.0 ± 16.3	71.5 ± 16.5	7d	THCQ + C	FR, AT, NS, OST	1, 2, 9
Lan et al. (2013)	China	28/32	13/15	15/17	78.17 ± 10.7	75.06 ± 11.2	7d	THCQ + C	FR, AT, NS, OST	2, 4, 7
Li et al. (2020)	China	19/20	12/7	11/9	66.0 ± 7.06	65.8 ± 3.11	7d	THCQ + C	AT, FR, VP, NS, Ipratropium bromide compound, Ambroxol	2, 4, 5, 6
Li (2023)	China	24/23	12/12	11/12	68.33 ± 12.23	66.65 ± 14.43	7d	THCQ + C	FR, AT, NS, OST	1, 2, 3, 4, 5, 6, 10
Shen et al. (2021)	China	57/57	33/24	31/26	65.43 ± 6.81	64.86 ± 6.29	NA	THCQ + C	FR, AT, VP, GC, NS, OST	1, 2, 3, 5, 6, 8
Shi (2012)	China	28/32	13/15	15/17	78.17 ± 10.7	75.06 ± 11.2	7d	THCQ + C	FR, AT, VP, IA, GC, OST	2, 4, 5, 6, 10
Wang et al. (2021)	China	30/30	15/15	17/13	62.00 ± 10.90	59.13 ± 11.79	7d	THCQ + C	FR, AT, VP, GC, NS, OST	1, 2, 4, 5, 6, 8, 12
Wang et al. (2024)	China	23/23	18/5	17/6	80.5 ± 11.7	78.6 ± 9.2	7d	THCQ + C	FR, AT, VP, GC, NS, OST	2, 3, 5, 6, 7, 11
Wu (2020)	China	25/25	14/11	15/10	50.9 ± 5.1	50.2 ± 4.9	14d	THCQ + C	AT, Ipratropium bromide compound, Ambroxol	4, 5, 6
Xie et al. (2023)	China	30/30	19/11	17/13	68.1 ± 7.3	66.6 ± 8.8	7d	THCQ + C	FR, IA, VP, GC, ACT, NS, AT, OST	1, 2, 4, 5, 6
Yang et al. (2009)	China	30/30	17/13	18/12	58.13 ± 11.9	61.36 ± 10.87	7d	THCQ + C	AT, OST	2, 10, 11
Zhang et al. (2021)	China	40/40	27/13	22/18	6.62 ± 1.14	6.35 ± 1.12	7d	THCQ + C	FR, AT, VP, GC, NS, OST	1, 2, 3, 7, 9
Zhu (2010)	China	24/26	13/11	16/10	70.04 ± 11.638	71.46 ± 11.819	7d	THCQ + C	GC, AT, OST	2, 4, 5, 10
Zhu et al. (2019)	China	30/30	16/14	17/13	63 ± 13.8	65 ± 12.5	5d	THCQ + C	AT, FR, VP, IA, GC, NS, OST	2, 4, 5

Annotation: NA: not applicable; C: Same conventional treatment as control group; FR: Fluid resuscitation; AT: Anti-infective therapy; VP: Vasopressors; IA: Inotropic agents; GC: Glucocorticoids; OST: Organ support therapy; NS: Nutritional support; ACT: Anticoagulant therapy; 1: 28-day mortality rate; 2: APACHE-Ⅱ; 3: SOFA; 4: WBC; 5: PCT; 6: CRP; 7: IL-6; 8: SAA; 9: endotoxin; 10: PLT; 11: D-II; 12: adverse events

## 2 Materials and methods

This systematic review was conducted referring to the Cochrane Handbook and reported according to the PRISMA-CHM check list (Supplementary Appendix S2) (Higgins et al., 2022). The protocol was registered in PROSPERO under registration number CRD42024562595.

### 2.1 Eligibility criteria

#### 2.1.1 Intervention

The intervention of THCQ was used as an adjunctive therapy to conventional medical treatment and was prepared by hospital or institution in a standardized process, regardless of dosage form, treatment duration, or administration route. We also included trials that assessed THCQ if the content of the formula was modified based on syndrome differentiation but still contained the following five main drugs: *P. persica* (L.) Batsch [Rosaceae; *Persicae Semen*] (Tao Ren), *R. palmatum* L. [Polygonaceae; *Rhei Radix et Rhizoma*] (Da Huang), *C. cassia* (L.) J. Presl [Lauraceae; *C. ramulus*] (Gui Zhi), *G. uralensis* Fisch. ex DC. [Fabaceae; *Glycyrrhizae Radix et Rhizoma*] (Gan Cao), and *Natrii Sulfas* [Sodium sulfate] (Mang Xiao). The composition of THCQ has been validated taxonomically using Plants of the World Online (POWO). All botanical drugs were selected and reported according to the guidelines of the “Consortium for Phytochemical Characterization of Medicinal Plants (ConPhyMP)” (Heinrich et al., 2022; Heinrich et al., 2020). Information on the drugs, dosage, and decoction preparation method of THCQ was given in Supplementary Appendix S1; Table1. The identified metabolites of THCQ was provided in Supplementary Appendix S1; Table 2.

**TABLE 2:** Risk of bias plot of the 16 RCTs.

Study ID	Randomization process	Deviations from intended interventions	Missing outcome data	Measurement of the outcome	Selection of the reported result	Overall
Bao (2013)	S	S	L	L	S	S
Chen et al. (2021)	S	S	L	L	S	S
Jin et al. (2020)	S	S	L	L	S	S
Lan et al. (2013)	L	S	L	L	S	S
Li et al. (2020)	S	S	L	L	S	S
Li (2023)	S	S	H	L	S	H
Shen et al. (2021)	S	S	L	L	S	S
Shi (2012)	S	S	L	L	S	S
Wang et al. (2021)	S	S	L	L	S	S
Wang et al. (2024)	L	S	L	L	S	S
Wu (2020)	S	S	L	L	S	S
Xie et al. (2023)	S	S	L	L	S	S
Yang et al. (2009)	S	S	L	L	S	S
Zhang et al. (2021)	S	S	L	L	S	S
Zhu (2010)	S	S	H	L	S	H
Zhu et al. (2019)	S	S	L	L	S	S

Annotation: L: low risk; H: high risk; S: some concerns.

#### 2.1.2 Participants

We included inpatient participants who had been diagnosed with sepsis according to specified diagnostic criteria in the guidelines. There were no restriction on age, race, gender, or disease duration of the patients. We included both children and adults diagnosed with sepsis based on Sepsis three criteria or the presence of at least two systemic inflammatory response syndrome (SIRS) symptoms: fever (body temperature >38 °C) or hypothermia (<36 °C), tachycardia (>90 beats per minute), tachypnea (>20 breaths per minute), hyperventilation (arterial carbon dioxide tension (PaCO2) < 32 mmHg), or abnormal white blood cell counts (>12,000 cells/mL or <4,000 cells/mL), with more than 10% immature neutrophils (Singer M. et al., 2016).

#### 2.1.3 Control

The control group received one or a combination of conventional medicine treatments. Conventional treatment typically included fluid resuscitation, anti-infective therapy, nutritional support, and organ support, among other interventions.

#### 2.1.4 Study designs to be included

Study types were limited to randomized controlled trials (RCTs).

#### 2.1.5 Main outcomes

The main outcomes were mortality rates, including 14-day and 28-day mortality rates. The 14-day or 28-day mortality rate referred to the proportion of patients who died within 14 or 28 days after their initial treatment. In clinical research, especially when evaluating severe diseases such as sepsis, researchers often use this measure to assess the efficacy of treatments. This time frame helped reflect the medium to long-term impact of the disease on patient survival outcomes (Laczynski D. J. et al., 2023).

#### 2.1.6 Other outcomes

Disease severity was assessed using the Acute Physiology and Chronic Health Evaluation II score (APACHE II) (LeGall J. R. et al., 1986), a widely accepted tool for evaluating the severity of illness in ICU patients. Organ function impairment was evaluated using Sequential Organ Failure Assessment score (SOFA) (Vincent J. L. et al., 1996), which reflects the extent of multiple organ failure, a hallmark of sepsis progression. Inflammation outcomes included Procalcitonin (PCT), C-reactive protein (CRP), endotoxin, white blood cell (WBC), serum amyloid A (SAA), and interleukin-6 (IL-6), as these are key indicators of systemic inflammation and infection severity in sepsis. Abnormal coagulation outcomes included platelets (PLT) and D-dimer, given that coagulation abnormalities frequently occur in septic patients and are associated with poor outcomes (Aklilu A. et al., 2025). Safety outcome included adverse events.

### 2.2 Search strategy

We searched the following electronic databases from their inception to 29 June 2024: PubMed, the Cochrane Library, EMBASE, Web of Science, China National Knowledge Infrastructure (CNKI), Wanfang database, Chinese Scientific Journal Database (VIP), and Chinese biomedical literature database (CBM). Details of the search strategy was shown in Supplementary Appendix S3; Table 1.

### 2.3 Research selection

After removing duplicate records, two authors independently conducted the initial screening of the studies based on titles and abstracts, followed by a full-text assessment to ensure relevance. Discrepancies were resolved through consensus, and a third author was consulted if necessary. Subsequently, two authors worked in pairs to extract data from the included studies using a predesignated extraction table to ensure consistency and accuracy.

### 2.4 Quality assessment

Two researchers independently assessed the quality of the RCTs. Any discrepancies were resolved through consensus, and a third author was consulted. The Cochrane Handbook’s Risk of Bias 2.0 tool was employed for quality assessment. The assessment included five domains: bias in the randomization process, bias in deviation from the intended intervention, bias in outcome measurement, bias in missing outcome data, and bias in selective outcome reporting.

### 2.5 Strategy of data synthesis

The statistical analysis was performed using RevMan software (version 5.4.1) and STATA software (version 17.0). For dichotomous variables, effect sizes were reported as Relative Risk (RR) with corresponding 95% confidence intervals (CI). Mean Difference (MD) with 95% CI was used for continuous variables, and the standardized mean difference (SMD) was used when the measurement units of outcomes differed. Heterogeneity among studies was assessed using the I^2^ test. A fixed-effect model was applied when heterogeneity was low (p > 0.1, I^2^ < 50%). However, if significant heterogeneity was detected (p < 0.1, I^2^ > 50%), a random-effects model was employed. Sensitivity analysis was performed for the primary outcome to assess the robustness of the conclusions. When severe heterogeneity existed, sensitivity and subgroup analysis were performed to explore potential sources of heterogeneity. Publication bias was assessed using Egger’s test and funnel plots. Conventional treatment (CT) referred to symptomatic therapies, whether used alone or in combination. These treatments were consolidated to better illustrate the overall difference in efficacy between THCQ and conventional therapy, making the results clearer.

### 2.6 Quality of evidence

Two authors independently assessed the certainty of each outcome using the Guideline Development Tool (GRADEpro GDT), developed by the Grading of Recommendations, Assessment, Development, and Evaluation (GRADE) working group (Guyatt G. H. et al., 2008). GDT was used to evaluate the certainty of evidence from five domains: risk of bias, directness, precision, consistency, and the possibility of publication bias.

## 3 Results

### 3.1 Database search

**FIGURE 1:**
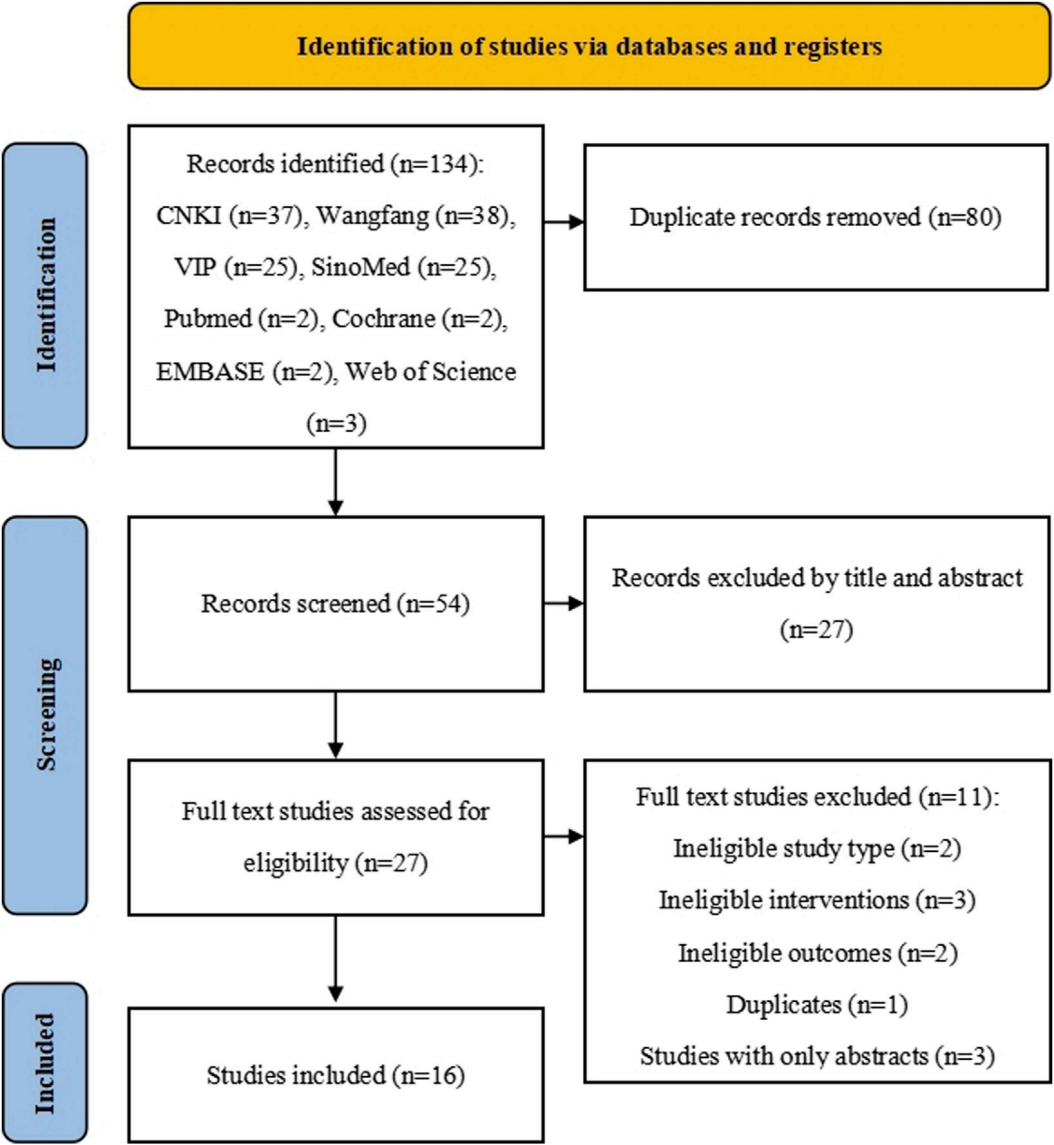
PRISMA flow chart of literature searching and screening.

A total of 134 records were retrieved from eight databases, with 80 duplicates removed. Initially, 27 studies were excluded by screening titles and abstracts. During the full-text screening process, 11 studies were excluded due to the inclusion criteria or duplicated. Ultimately, 16 RCTs were included, Figure 1.

### 3.2 Study characteristics

Sixteen RCTs were published between 2009 and 2024, all conducted in China, involving patients aged 6–80 years. A total of 1,034 sepsis patients were included, with 511 in the experimental group and 523 in the control group. The treatment duration ranged from 5 to 14 days, six studies used modified THCQ. Conventional treatments varied across 16 studies, with most including fluid resuscitation, anti-infective therapy, glucocorticoids, and nutritional support. The detailed characteristics of the included trials are shown in Table 1. The specific dosages of each drug of THCQ used in the studies were detailed in Supplementary Appendix S1; Table 3. ConPhyMP checklist was shown in Supplementary Appendix S6.

**TABLE 3:** Efficacy and safety of THCQ as an adjuvant therapy for sepsis.

Outcomes	No. of studies	No. of participants	Effect model (R/F)	I^2^ (%)	Type of effect size	Effect size [95%CI]	Quality of evidence (GRADE)
THCQ + CT *versus* CT							
28-day mortality rate	6	429	F	0	RR	0.57 [0.38, 0.86]	Low^1,3^
APACHE-Ⅱ score	14	919	R	72	MD	−2.37 [-3.12, −1.63]	Low^1,4^
SOFA score	5	407	R	79	MD	−1.41 [-2.12, −0.70]	Low^1,4^
WBC (10^9^/L)	10	541	R	88	MD	−1.78 [-2.97, −0.59]	Low^1,4^
PCT (ng/mL)	11	706	R	95	MD	−1.20 [-1.71, −0.69]	Low^1,4^
CRP (mg/L)	9	596	R	84	MD	−9.82 [-13.98, −5.66]	Low^1,4^
IL-6 (pg/mL)	4	301	R	93	MD	−33.55 [-52.77, −14.33]	Very Low^1,2,4^
SAA (mg/L)	2	174	F	0	MD	−11.10 [-15.57, −6.62]	Low^1,2^
Endotoxin (eU/mL)	2	148	R	91	SMD	−0.76 [-1.92, 0.41]	Very Low^1,2,4^
PLT (10^9^/L)	5	277	R	44	MD	23.42 [11.71, 35.14]	Low^1,2^
D-II (mg/L)	3	166	R	57	MD	−0.68 [-1.71, 0.35]	Very Low^1,2,4^
Adverse events	1	60	NA	NA	RR	0.67 [0.12, 3.71]	Very Low^1,2,3^

Annotation: NA: not applicable; F: fixed-effects model; R: randomized-effects model; RR: risk ratio; MD: weighted mean difference; SMD: standardized mean difference; 1: serious risk of bias, lack of description of blinding; 2: serious imprecision due to insufficient sample size; 3: serious imprecision due to the infrequency of events; 4: serious inconsistency due to obvious heterogeneity

### 3.3 ROB 2.0 analysis

**FIGURE 2:**
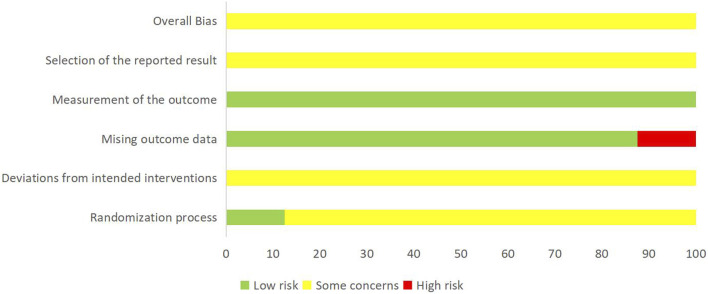
Summary of risk of bias in the included studies.

The risk of bias assessment of the 16 RCTs was summarized in Table 2; Figure 2. The results showed that 14 RCTs were assessed as having some concerns, while 2 RCTs were assessed as having high risk of bias. Regarding the randomization process, 14 studies used random number tables to generate random sequences, and two studies used opaque envelopes for random sequence allocation. In terms of deviations from intended interventions and outcome measurement, all studies combined THCQ with conventional treatments without a placebo-controlled design. For missing outcome data, two studies had a dropout rate of over 15%, resulting in a high risk rating. Regarding statistical analysis, all 16 studies detailed their methods and were rated as low risk. None of the studies were registered.

### 3.4 Mortality rate

**FIGURE 3:**
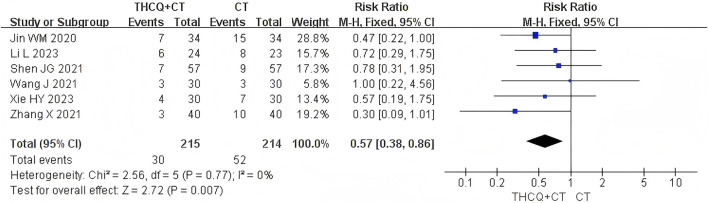
Forest plots of the comparison on 28-day mortality rate.

Among the 16 included studies, six used the 28-day mortality rate as an outcome, no studies used the 14-day mortality rate as an outcome. Meta-analysis showed that the combination of THCQ with CT reduced the 28-day mortality rate compared to CT alone (RR = 0.57, 95% CI: 0.38 to 0.86; six trials, 429 participants, I^2^ = 0%), as shown in Table 3, Figure 3. Subgroup analysis based on age revealed that, for the 28-day mortality rate, THCQ combined with CT was superior than CT alone in adults (RR = 0.64, 95% CI: 0.42 to 0.98; five trials, 349 participants, I^2^ = 0%). However, in children, no statistically significant difference was observed between the two groups (RR = 0.30, 95% CI: 0.09 to 1.01; one trial, 80 participants). Furthermore, subgroup analysis suggests that THCQ in its original formula combined with CT was still superior than CT alone in reducing the 28-day mortality rate (RR = 0.50, 95% CI: 0.27 to 0.94; two trials, 128 participants, I^2^ = 0%), Table 4.

**TABLE 4:** Subgroup analysis of THCQ as an adjuvant therapy for sepsis.

Outcomes	Subgroup	No. of studies	No. of participants	Effect model (R/F)	I^2^ (%)	Type of effect size	Effect size [95%CI]
THCQ + CT *versus* CT							
28-day mortality rate	Adult	5	349	F	0	RR	0.64 [0.42, 0.98]
	Child	1	80	NA	NA	RR	0.30 [0.09, 1.01]
APACHE-Ⅱ score	Adult	13	839	R	74	MD	−2.40 [-3.22, −1.58]
	Child	1	80	NA	NA	MD	−2.29 [-3.48, −1.10]
SOFA score	Adult	4	327	R	81	MD	−2.04 [-3.38, −0.70]
	Child	1	80	NA	NA	MD	−0.73 [-0.99, −0.47]
IL-6 (pg/mL)	Adult	3	221	R	94	MD	−33.50 [-61.52, −5.47]
	Child	1	80	NA	NA	MD	−35.27 [-45.20, −25.34]
28-day mortality rate	Original THCQ	2	128	F	0	RR	0.50 [0.27, 0.94]
	Modified THCQ	4	429	F	0	RR	0.63 [0.37, 1.06]
APACHE-Ⅱ score	Original THCQ	8	452	R	22	MD	−1.55 [-2.10, −1.00]
	Modified THCQ	6	467	R	80	MD	−3.59 [-5.10, −2.09]
SOFA score	Modified THCQ	5	407	R	79	MD	−1.41 [-2.12, −0.70]
WBC (10^9^/L)	Original THCQ	8	434	R	88	MD	−1.52 [-2.80, −0.23]
	Modified THCQ	2	107	R	80	MD	−2.88 [-5.62, −0.15]
SAA (mg/L)	Modified THCQ	2	174	F	0	MD	−11.10 [-15.57, −6.62]
CRP (mg/L)	Original THCQ	4	209	R	91	MD	−7.80 [-16.66, 1.05]
	Modified THCQ	5	387	R	73	MD	−10.52 [-15.34, −5.71]
PCT (ng/mL)	Original THCQ	6	319	R	96	MD	−1.22 [-1.83, −0.61]
	Modified THCQ	5	387	R	79	MD	−1.18 [-1.93, −0.42]
PLT (10^9^/L)	Original THCQ	4	230	R	19	MD	20.51 [13.79, 27.24]
	Modified THCQ	1	47	NA	NA	MD	68.73 [18.68, 118.78]
IL-6 (pg/mL)	Original THCQ	1	55	NA	NA	MD	−47.64 [-58.46, −36.82]
	Modified THCQ	3	246	R	85	MD	−26.48 [-43.84, −9.11]
D-II (mg/L)	Original THCQ	2	120	R	75	MD	−0.65 [-1.95, 0.65]
	Modified THCQ	1	46	NA	NA	MD	−1.20 [-3.64, 1.24]
Endotoxin (eU/mL)	Original THCQ	1	68	NA	NA	SMD	−0.16 [-0.64, 0.31]
	Modified THCQ	1	80	NA	NA	SMD	−1.35 [-1.84, −0.86]

Annotation: NA: not applicable; F: fixed-effects model; R: randomized-effects model; RR: risk ratio; MD: weighted mean difference; SMD: standardized mean difference.

### 3.5 Disease severity outcome

Fourteen studies reported APACHE-II scores, and the pooled results suggested that the combination of THCQ with CT was superior to CT alone in improving the severity of sepsis (MD = −2.37, 95% CI: −3.12 to −1.63; 14 trials, 919 participants, I^2^ = 72%). Subgroup analysis showed that, for APACHE-II scores, THCQ combined with CT was superior to CT alone in both adults (MD = −2.40, 95% CI: −3.22 to −1.58; 13 trials, 839 participants, I^2^ = 74%) and children (MD = −2.29, 95% CI: −3.48 to −1.10; one trial, 80 participants). In addition, THCQ in its original formula combined with CT was still superior than CT alone in improving APACHE-II score (MD = −1.55, 95% CI: −2.10 to −1.00; eight trials, 452 participants, I^2^ = 22%), Table 4. The forest plot was shown in Supplementary Appendix S4; Figure 1.

### 3.6 Organ function impairment outcome

Five studies reported SOFA scores, indicating that the combination of THCQ with CT had a statistically significant advantage over CT alone in improving the severity of organ dysfunction in sepsis patients (MD = −1.41, 95% CI: −2.12 to −0.70; five trials, 407 participants, I^2^ = 79%). Additionally, subgroup analysis showed that the combined of THCQ improved the severity of organ dysfunction in both adult (MD = −2.04, 95% CI: −3.38 to −0.70; four trials, 327 participants, I^2^ = 81%) and pediatric sepsis patients (MD = −0.73, 95% CI: −0.99 to −0.47; one trial, 80 participants), Table 3, 4. The forest plot was shown in Supplementary Appendix S4 Figure 1.

### 3.7 Inflammation outcomes

As shown in Table 3, fourteen studies reported white blood cell counts, and the pooled results suggested that the combination of THCQ with CT resulted in lower WBC levels in sepsis patients compared to CT alone (MD = −1.78 10^9^/L, 95% CI: −2.97 to −0.59; 10 trials, 541 participants, I^2^ = 88%). Eleven studies reported procalcitonin levels, indicating that THCQ combined with CT had a statistically significant advantage over CT alone in lowering PCT levels in sepsis patients (MD = −1.20 ng/mL, 95% CI: −1.71 to −0.69; 11 trials, 706 participants, I^2^ = 95%). Ten studies reported C-reactive protein levels, and the pooled results suggested that the combination of THCQ with CT was superior to CT alone in lowering CRP levels in sepsis patients (MD = −9.82 mg/L, 95% CI: −13.98 to −5.66; nine trials, 596 participants, I^2^ = 84%). Four studies reported interleukin-6 levels, showing that the combination of THCQ and CT had some advantage in reducing IL-6 levels in sepsis patients, with a statistically significant difference (MD = −33.55 pg/mL, 95% CI: −52.77 to −14.33; four trials, 301 participants, I^2^ = 93%). Two studies reported serum amyloid A levels, with pooled results indicating that THCQ combined with CT resulted in lower SAA levels compared to CT alone (MD = −11.10 mg/L, 95% CI: −15.57 to −6.62; two trials, 174 participants, I^2^ = 0%). For endotoxin, two studies suggested that THCQ combined with CT was not superior to CT alone in reducing endotoxin levels, with no statistically significant difference observed (SMD = −0.76 eU/mL, 95% CI: −1.92 to 0.41; two trials, 148 participants, I^2^ = 91%). Subgroup analysis indicated that, compared to CT alone, the combination of THCQ had some advantage in reducing IL-6 levels in both adult (MD = −33.50, 95% CI: −61.52 to −5.47; three trials, 221 participants, I^2^ = 94%) and pediatric (MD = −35.27, 95% CI: −45.20 to −25.34; one trial, 80 participants for children) sepsis patients. Furthermore, THCQ in its original formula combined with CT also showed some advantages in improving WBC, PCT, and IL-6 levels, Table 4. The forest plot was shown in Supplementary Appendix S4; Figure 2.

### 3.8 Coagulation outcomes

Five studies reported platelet counts, and the pooled results suggested that the combination of THCQ with CT was superior to CT alone in improving PLT levels in sepsis patients (MD = 23.42 10^9^/L, 95% CI: 11.71 to 35.14; five trials, 277 participants, I^2^ = 44%). Three studies reported D-dimer levels, indicating that THCQ combined with CT was not superior to CT alone in reducing D-dimer levels, with no statistically significant difference observed (MD = −0.68 mg/L, 95% CI: −1.71 to 0.35; three trials, 166 participants, I^2^ = 57%), Table 3. The forest plot was shown in Supplementary Appendix S4; Figure 3. Subgroup analysis showed that, THCQ in its original formula combined with CT was still superior than CT alone in improving PLT level (MD = 20.51, 95% CI: 13.79 to 27.24; four trials, 230 participants, I^2^ = 19%), Table 4.

### 3.9 Adverse events

Most RCTs on THCQ did not report any serious adverse events. One study (Wang. et al., 2021) reported that the experimental group received modified THCQ in combination with treatments such as fluid resuscitation, anti-infective therapy, and mechanical ventilation. During the treatment course, two cases of mild abdominal distension and diarrhea occurred in the experimental group, and three cases occurred in the control group. All symptoms resolved after symptomatic treatment, and no gastrointestinal mucosal bleeding events were observed.

### 3.10 Sensitivity analysis

**FIGURE 4:**
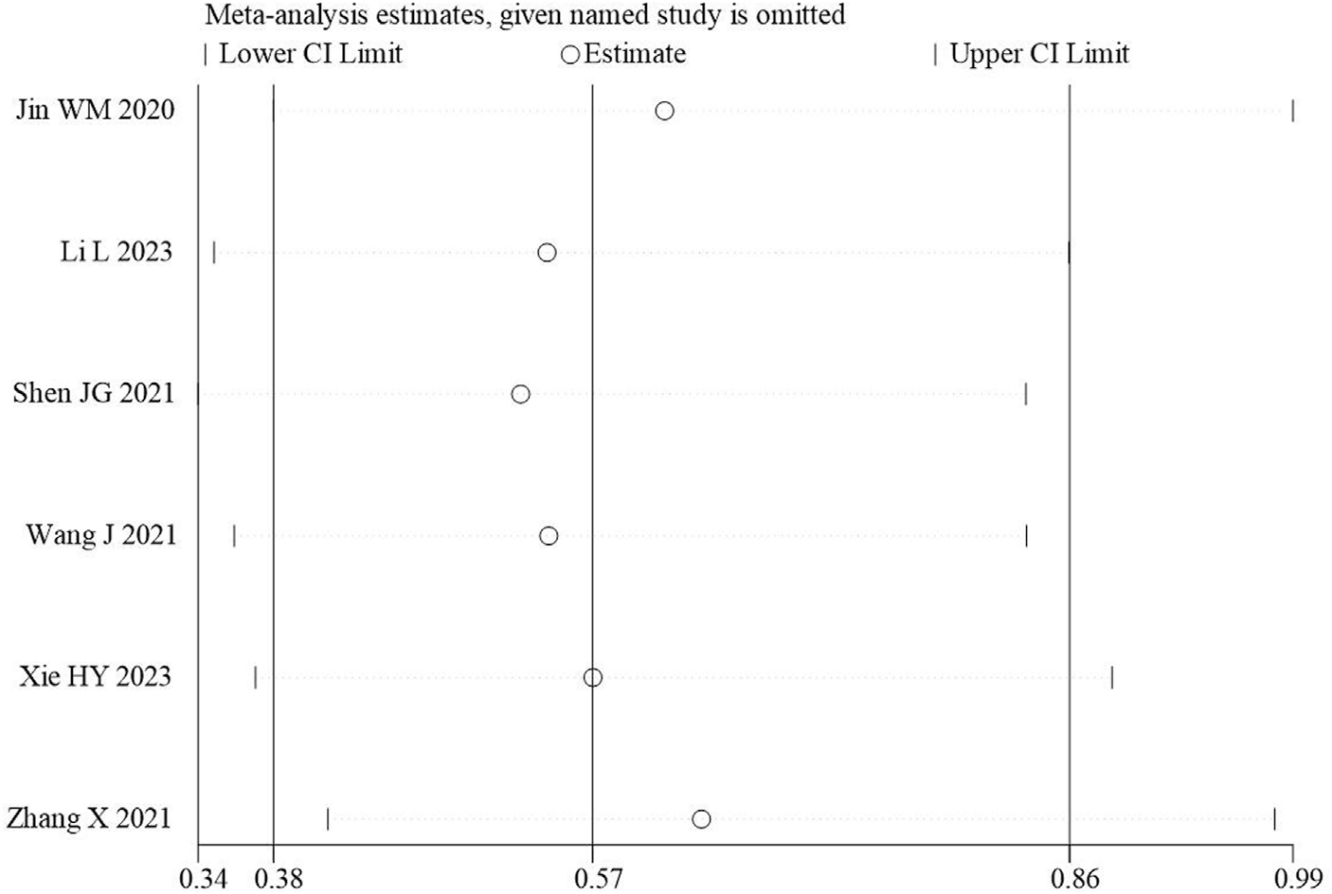
Sensitivity analysis of 28-day mortality rate.

The sensitivity analysis on the primary outcome showed when (Jin et al., 2020) or (Zhang and Han, 2010) was excluded, the confidence interval widened, indicating some influence from the study. However, the confidence interval did not cross the line of no effect, suggesting that the overall results remain robust, which indicated that the pooled effects of primary outcomes in the comparison between THCQ plus CT and CT were robust, Figure 4. We also conducted sensitivity analyses on APACHE-Ⅱ, SOFA, WBC, PCT, CRP, IL-6, PLT, and the results remained robust (Supplementary Appendix S5). Sensitivity analysis for the other outcome measures was not feasible due to limited number of included studies.

### 3.11 Publication bias assessment

We conducted publication bias analysis for outcomes that included more than 10 studies. The distribution of scattered points in the funnel plots was generally symmetrical. Egger’s test for funnel plot asymmetry indicated that there was almost no publication bias for WBC and PCT in the included studies (P = 0.993, P = 0.781). But APACHE-Ⅱ may be influenced by selective reporting of studies (P = 0.023). (Supplementary Appendix S5).

### 3.12 Certainty of evidence

Table 3 outlines the certainty of evidence along with the reasons for any adjustments. All outcomes were evaluated as having low to very low certainty, primarily due to issues related to risk of bias, imprecision, inconsistency, and indirectness.

## 4 Discussion

### 4.1 Findings from systematic review

This study aims to provide further insights into the potential and safety of THCQ as an adjunctive therapy for sepsis. Through a systematic review of eight databases, 16 RCTs involving a total of 1,034 sepsis patients were included. Our meta-analysis found that THCQ may reduce the 28-day mortality rate in sepsis patients (RR = 0.57, 95% CI: 0.38–0.86), as well as improve APACHE-II and SOFA scores. Additionally, THCQ appears to affect levels of WBC, PCT, CRP, IL-6, SAA, and PLT, with no severe adverse events observed, providing a valuable strategy for the treatment of sepsis.

### 4.2 Potential protective effects of THCQ

Sepsis is a severe condition characterized by organ dysfunction resulting from an uncontrolled host response to infection (Singer M. et al., 2016). And inflammatory and coagulation markers are crucial for the diagnosis and monitoring of sepsis, especially in emergency settings, as they provide a more accurate depiction of the patient’s current condition and guide clinical decisions (Rivers E. et al., 2001). Coagulation dysfunction is a prominent feature of sepsis and is closely related to inflammation. Thrombocytopenia or low platelet count is associated with higher mortality and poorer prognosis in sepsis patients (Huang C. et al., 2023). In our meta-analysis, THCQ showed some improvement in inflammatory and coagulation markers. Some studies have found that THCQ offers protective effects against sepsis-associated acute lung injury (ALI) and sepsis-induced cardiac dysfunction (SICD) in cellular and animal models. THCQ reduces key inflammatory factors in sepsis, such as TNF-α, IL-1β, and IL-6, inhibits oxidative stress, and alleviates iron overload, indicating its multiple protective mechanisms in mitigating the inflammatory cascade of sepsis (Deng M. et al., 2024; Lu S. M. et al., 2024; Han X. et al., 2023). Additionally, THCQ has been found to regulate critical target genes in sepsis patients (such as MAPK14, MAPK3, MMP9, STAT3, and LYN), which play key roles in cellular inflammatory responses, especially through the modulation of the MAPK and Nrf2 pathways (Deng M. et al., 2024; Lu S. M. et al., 2024).

Network pharmacology findings further support the theoretical basis for THCQ as a potentially effective treatment. Multiple active metabolites of THCQ, such as the anti-inflammatory metabolites from *P. persica* (L.) Batsch [Rosaceae; *Persicae Semen*], emodin from *R. palmatum* L. [Polygonaceae; *Rhei Radix et Rhizoma*], 6-gingerol from *C. cassia* (L.) J. Presl [Lauraceae; *C. ramulus*], and the immunomodulatory metabolites from *G. uralensis* Fisch. ex DC. [Fabaceae; *Glycyrrhizae Radix et Rhizoma*], may work synergistically to exert therapeutic effects (Seo K. H. et al., 2020; Huang Q. et al., 2007; Ma X. et al., 2016; Chen I. C. et al., 2018; Zhou S. et al., 2020). Moreover, we found that THCQ may reduce the 28-day mortality rate in sepsis patients, which is a valuable clinical finding for patients. The 28-day mark is a critical time point for evaluating the efficacy of acute phase treatments for sepsis, and reducing mortality during this period means that more patients can survive the acute crisis, allowing valuable time for subsequent rehabilitation (Evans L. et al., 2021).

Additionally, according to the theory of pattern identification in TCM, THCQ is particularly suitable for sepsis patients exhibiting TCM patterns such as “blood stasis,” “fire toxin,” and “qi impediment,” which is also reflected in the original studies included in our analysis. Specifically, *P. persica* (L.) Batsch [Rosaceae; *Persicae Semen*] and *C. cassia* (L.) J. Presl [Lauraceae; *C. ramulus*] help alleviate “blood stasis” by promoting blood circulation; *R. palmatum* L. [Polygonaceae; *Rhei Radix et Rhizoma*] and *Natrii Sulfas* [Sodium sulfate] clear “fire toxin” and eliminate dampness, thereby unblocking “qi impediment” to promote the smooth flow of qi and blood (He S. P. et al., 2004). This approach aligns with TCM’s holistic treatment method, aiming to restore the body’s balance and enhance its self-healing ability, thereby improving the overall therapeutic effect in sepsis patients. Thus, THCQ offers a novel strategy for managing emergency and severe conditions in TCM, providing a new approach for the rescue and long-term rehabilitation of sepsis patients, as supported by experimental research, TCM theory, and clinical practice.

### 4.3 Strengths and limitations

To the best of our knowledge, this study is the first systematic review evaluating the adjunctive use of THCQ in the treatment of sepsis patients. We conducted a systematic review and meta-analysis according to a pre-registered study protocol, employing a comprehensive search strategy to ensure that no relevant RCTs were overlooked. Most of the studies included in our review were published in the past 5 years, and each study was assessed using the ROB 2.0 tool to ensure methodological rigor. Additionally, to guarantee the robustness of our results, we performed detailed sensitivity analyses and publication bias assessments.

However, several limitations should be considered when interpreting the results of this meta-analysis. Firstly, all included studies were conducted in China with predominantly Asian participants, which may limit the generalizability of the findings to non-Asian populations. Secondly, the methodological quality of the included studies was generally low, with most lacking pre-registered protocols, blinding, and placebo controls. These shortcomings may introduce bias and reduce the internal validity and reproducibility of the results. Thirdly, given the individualized nature of sepsis treatment, we categorized the control groups under “conventional treatment” to better illustrate the efficacy difference between THCQ and CT. However, we recognize that differences in treatment protocols may affect the comparability and interpretation of the results. Future studies should provide more detailed treatment protocols for the control groups and perform subgroup analyses to more accurately assess the effects of different interventions. Moreover, our meta-analysis revealed significant heterogeneity across several outcomes. To explore potential sources of this heterogeneity, we performed subgroup analyses based on use of modified THCQ, types of CT in the control group, patient age, sepsis type, and duration of treatment. However, heterogeneity within the subgroups did not significantly decrease. Therefore, given the lack of consistency between studies, the findings of this meta-analysis should be interpreted with caution.

### 4.4 Implications for research

Based on the current research findings, THCQ may offer additional survival benefits for sepsis patients. However, the existing evidence still carries uncertainty. Therefore, how to effectively integrate THCQ into the current sepsis management guidelines requires further large-scale, multicenter trials to better establish its efficacy and safety in clinical practice. Considering the uncertainty of the evidence, future research must prioritize methodological rigor and transparency. To improve trial quality, appropriate allocation concealment must be implemented, and blinding of participants, researchers, and outcome assessors should be ensured. Future trial reports should adhere to international standards, such as the CONSORT statement, and trial protocols should be registered and publicly available. Additionally, future studies should evaluate the quality of the botanical drugs based on established standards, specifically providing detailed information on botanical drug species, geographical origin, harvest season, preparation methods, and product quality to ensure reproducibility and standardization. Given the severity of sepsis, future research should also focus on survival rates at different treatment time points to reflect THCQ’s applicability during various stages of sepsis recovery. Of particular importance is that the existing research provides very limited reporting on the safety of THCQ. Given that sepsis is a condition with high morbidity and mortality, future studies should place greater emphasis on the adverse effects of THCQ, especially long-term adverse events, and incorporate long-term and systematic adverse event monitoring to ensure that its safety is thoroughly documented and reported.

## 5 Conclusion

In conclusion, our systematic review found that THCQ, as a representative of classic traditional Chinese medicine, may improve the 28-day mortality rate, APACHE-II score, SOFA score, inflammatory markers, and coagulation function in sepsis patients when combined with conventional treatment, without serious adverse events observed. However, given the low quality of the included trials, caution are needed in interpreting these results. Rigorous, multicenter, large-scale, and methodologically sound RCTs are needed.

## Data Availability

The original contributions presented in the study are included in the article/Supplementary Material, further inquiries can be directed to the corresponding author.
