# Supplementary material for: Classical formula Taohe Chengqi decoction as an adjuvant therapy for sepsis - a systematic review and meta-analysis of randomized controlled trials
Source: Front Pharmacol. 2025 Sep 2;16:1499280. doi: 10.3389/fphar.2025.1499280 (PMC12436689; doi:10.3389/fphar.2025.1499280)
Supplement: Supplementary file 1 [file Supplementaryfile1.docx]

**Table 1 Basic information of drug composition in THCQ.**

| **Common**  **English**  **name** | **Latin name** | **Chinese name** | **Authorities** | **Family** | **Genus** | **Medicinal part** | **Preparation method** | **Dosage (grams)** | **Main bioactive compounds** | **Medicinal source (Pharmacopoeia)** |
| --- | --- | --- | --- | --- | --- | --- | --- | --- | --- | --- |
| Peach Kernel | *Prunus persica* (L.) Batsch | Tao Ren桃仁 | Carl Linnaeus | Rosaceae | Prunus | Dried ripe seed | Remove impurities and crush when in use | 12 | Amygdalin, Amygdalin Saponin, Quercetin | China Pharmacopoeia (2020) |
| Rhubarb | *Rheum palmatum* L. | Da Huang大黄 | Carl Linnaeus | Polygonaceae | Rheum | Dried root and rhizome | Remove impurities, wash thoroughly, moisten until softened, cut into thick slices or chunks, and dry | 12 | Anthraquinones, Rhein, Chrysophanol, Emodin-8-glucoside, Aloe-emodin | European Pharmacopoeia, 7th edn. (2012) |
| Cinnamon Twig | *Cinnamomum cassia* (L.) J. Presl | Gui Zhi桂枝 | Carl Linnaeus | Lauraceae | Cinnamomum | Dried stem bark | Remove impurities, wash clean, moisten thoroughly, cut into thick slices, and dry. | 6 | Cinnamaldehyde, Quercetin, Isoquercetin, Coumarin | China Pharmacopoeia (2020) |
| Sodium sulfate | *Natrii Sulfas* | Mang Xiao芒硝 | NA | NA | NA | Mineral | Mining, wash clean, purify, and heat | 6 | Sodium sulfate decahydrate | China Pharmacopoeia (2020) |
| Licorice | *Glycyrrhiza uralensis* Fisch. ex DC. | Gan Cao甘草 | Friedrich Ernst Ludwig von Fischer | Fabaceae | Glycyrrhiza | Dried root and rhizome | Remove impurities, wash clean, and dry | 6 | Glycyrrhizin, glycyrrhinic acid, glycyrrhetic acid, liquiritin, liquiritigenin, glycyamarin, iso-liquiritin, dihydroxyglycyrrhetic acid, licoricidin, glycyrol, 5-0-methyl glycerol, iso-glycyrol | China Pharmacopoeia (2020) |

Annotation: According to the "Chinese Pharmacopoeia," the preparation method for traditional Chinese medicine (TCM) decoction is as follows: Weigh the following medicinal materials according to the prescribed dosage (grams): *Prunus persica* (L.) Batsch [Rosaceae; *Persicae Semen*], *Rheum palmatum* L. [Polygonaceae; *Rhei Radix et Rhizoma*], *Cinnamomum cassia* (L.) J.Presl [Lauraceae; *Cinnamomi ramulus*], *Natrii Sulfas* [Sodium sulfate], *Glycyrrhiza uralensis* Fisch. ex DC. [Fabaceae; *Glycyrrhizae Radix et Rhizoma*]. Remove impurities and thoroughly clean the substances. Place them into a clean container, add sufficient water, and soak for about 30 minutes until softened. Transfer the substances and soaking liquid to a ceramic or stainless steel pot, add 6–10 times the weight of water relative to the substances, bring to a boil, then simmer on low heat for 30 minutes. After the first decoction, filter the liquid and add water to the residue for a second decoction for 20–25 minutes. Mix the two decoctions thoroughly, then filter again through a fine sieve or gauze to clarify. While hot, transfer the decoction into a clean container, seal it, and store. Consume within 24 hours. For long-term storage, the decoction should be reboiled for sterilization and then refrigerated. It should be consumed warm in divided doses, with the dosage and frequency adjusted according to medical advice.

**Table 2 Metabolites identified of THCQ by HPLC-Q-Exactive-MC**

| **No** | **Name** | **PubChem CID** | **Rt/min** | **Formula** | **Weight** | **Calc. MW** | **Error (Da)** | **M/Z** | **Type** |
| --- | --- | --- | --- | --- | --- | --- | --- | --- | --- |
| 1 | D(-)-Quinic Acid | 6508 | 0.84 | C7H12O6 | 192.17 | 191.05501 | 1.547 | 191.05531 | Organic acids |
| 2 | Fumaric acid | 444972 | 0.85 | C4H4O4 | 116.07 | 115.00258 | -2.218 | 115.00233 | Organic acids |
| 3 | arginine | 6322 | 0.86 | C6H14N4O2 | 174.2 | 175.11895 | 0.387 | 175.11902 | Amino acids |
| 4 | Valine | 6287 | 0.91 | C5H11NO2 | 117.15 | 118.08625 | 1.227 | 118.0864 | Amino acids |
| 5 | malic acid | 525 | 0.93 | C4H6O5 | 134.09 | 133.01314 | -0.524 | 133.01308 | Organic acids |
| 6 | citric acid | 311 | 1.41 | C6H8O7 | 192.12 | 191.01862 | 1.68 | 191.01895 | Organic acids |
| 7 | Succinic acid | 1110 | 1.6 | C4H6O4 | 118.09 | 117.01823 | -1.924 | 117.01801 | Organic acids |
| 8 | Gallic acid | 370 | 2.98 | C7H6O5 | 170.12 | 169.01314 | 0.001 | 169.01315 | Organic acids |
| 9 | Gallic acid-O-diglucoside | 11504173 | 5.05 | C19H26O15 | 332.26 | 493.11879 | 3.86 | 493.1207 | Flavone |
| 10 | protocatechuic acid | 72 | 5.78 | C7H6O4 | 154.12 | 153.01823 | 1.665 | 153.0182 | Organic acids |
| 11 | 4-Hydroxybenzoic acid | 135 | 10.04 | C7H6O3 | 138.12 | 137.02332 | -0.296 | 137.02328 | Organic acids |
| 12 | Methyl gallate | 7428 | 11.21 | C8H8O5 | 184.15 | 183.02879 | 1.531 | 183.02908 | tannins |
| 13 | Catechin | 9064 | 11.91 | C15H14O6 | 290.27 | 289.07066 | 5 | 289.07211 | Flavone |
| 14 | Amygdalin | 656516 | 12.57 | C20H27NO11 | 457.43 | 502.15551 | -1.446 | 502.15479 | Flavone |
| 15 | Caffeic acid | 689043 | 13.26 | C9H8O4 | 180.16 | 179.03388 | 1.535 | 179.03416 | Organic acids |
| 16 | Hastatoside | 92043450 | 13.88 | C17H24O11 | 404.37 | 403.12348 | 3.379 | 403.12485 | Flavone |
| 17 | Bergapten | 2355 | 16.45 | C12H8O4 | 216.19 | 217.04953 | -1.775 | 217.04915 | Flavone |
| 18 | ferulic acid | 445858 | 16.85 | C10H10O4 | 194.18 | 193.04953 | 1.889 | 193.0499 | Organic acids |
| 19 | Lindleyin | 42994 | 17.22 | C23H26O11 | 478.45 | 477.13913 | 3.043 | 477.14059 | Flavone |
| 20 | Liquiritin | 503737 | 17.45 | C21H22O9 | 418.39 | 417.118 | -2.106 | 417.11713 | Flavone |
| 21 | Liquiritin apioside | 10076238 | 17.7 | C26H30O13 | 550.51 | 549.16026 | 3.137 | 549.16199 | Flavone |
| 22 | Liquiritigenin | 114829 | 17.76 | C15H12O4 | 256.25 | 257.08083 | -1.032 | 257.08057 | Flavone |
| 23 | Vitexin-2''-O-rhamnoside | 5282151 | 19.19 | C27H30O14 | 578.52 | 577.15518 | 2.596 | 577.15668 | Flavone |
| 24 | Ellagic acid | 5281855 | 19.73 | C14H6O8 | 302.19 | 300.99789 | 4.473 | 300.99924 | tannins |
| 25 | Rhein-8-glucoside | 5320961 | 20.1 | C21H18O11 | 446.36 | 445.07653 | 3.78 | 445.07822 | Flavone |
| 26 | Isoliquiritigenin | 638278 | 20.44 | C15H12O4 | 256.25 | 257.08083 | -1.032 | 257.08057 | Flavone |
| 27 | Isoliquiritoside | 5318591 | 20.68 | C21H22O9 | 418.39 | 417.118 | 3.959 | 417.11966 | Flavone |
| 28 | Isorhamnetin | 5281654 | 21.65 | C16H12O7 | 316.26 | 315.04992 | 4.351 | 315.0513 | Flavone |
| 29 | Hispidulin | 5281628 | 21.7 | C16H12O6 | 300.26 | 299.05501 | 4.198 | 299.05627 | Flavone |
| 30 | Naringenin | 439246 | 22.05 | C15H12O5 | 272.25 | 271.06009 | 5.239 | 271.06152 | Flavone |
| 31 | emodin-O-di-glucoside | 71587230 | 22.22 | C27H30O15 | 594.5 | 593.15009 | 3.277 | 593.15204 | Flavone |
| 32 | Quercetin | 5280343 | 22.26 | C15H10O7 | 302.24 | 301.03427 | 4.753 | 301.03571 | Flavone |
| 33 | Licochalcone B | 5318999 | 22.61 | C16H14O5 | 286.28 | 287.0914 | -2.125 | 287.09079 | Flavone |
| 34 | Chrysophanol 8-O-glucoside | 442731 | 22.75 | C21H20O9 | 416.38 | 415.10235 | 3.81 | 415.10394 | Flavone |
| 35 | Medicarpin | 336327 | 23.23 | C16H14O4 | 270.28 | 271.09648 | -2.565 | 271.09579 | Flavone |
| 36 | Luteolin-7-O-glucoside | 5280637 | 23.26 | C21H20O11 | 448.38 | 447.09218 | 3.561 | 447.09378 | Flavone |
| 37 | Emodin-8-beta-D-glucoside | 99649 | 23.79 | C21H20O10 | 432.38 | 431.09727 | 3.287 | 431.09869 | Flavone |
| 38 | Rhein | 10168 | 24.47 | C15H8O6 | 284.22 | 283.02371 | 5.072 | 283.02515 | anthraquinones |
| 39 | Dihydroxy glycyrrhiza hypoic acid | 114585 | 24.52 | C42H62O18 | 470.68 | 853.38524 | 3.666 | 853.38837 | triterpenes |
| 40 | Chrysophanic acid | 10208 | 24.75 | C15H10O4 | 254.24 | 253.04953 | 4.326 | 253.05063 | anthraquinones |
| 41 | Formononetin | 5280378 | 24.86 | C16H12O4 | 268.26 | 269.08083 | 0.017 | 269.08084 | Flavone |
| 42 | Licoricesaponin H | 101589724 | 24.99 | C42H60O16 | 822.93 | 819.37976 | 2.609 | 819.3819 | saponins |
| 43 | Luteolin | 5280445 | 25.31 | C15H10O6 | 286.24 | 285.03936 | 4.826 | 285.04074 | Flavone |
| 44 | Calycosin | 5280448 | 25.35 | C16H12O5 | 284.26 | 285.07575 | 0.211 | 285.07581 | Flavone |
| 45 | Physcion 8-O-beta-D-monoglucoside | 168938 | 25.46 | C22H22O10 | 446.4 | 445.11292 | 3.879 | 445.11465 | Flavone |
| 46 | Rhein 8-b-D-Glucuronide | 156112 | 25.6 | C21H16O_1_2 | 460.34 | 297.03936 | 3.621 | 297.04044 | anthraquinones |
| 47 | Neoglycyrol | 5320083 | 25.63 | C21H18O6 | 366.36 | 367.11761 | -0.503 | 367.11743 | Flavone |
| 48 | Licoricesaponin A3 | 14187172 | 25.79 | C48H72O21 | 985.07 | 983.44823 | 2.862 | 983.45105 | saponins |
| 49 | sigmoidin B | 73205 | 25.82 | C20H20O6 | 356.37 | 357.13326 | -1.022 | 357.1329 | Flavone |
| 50 | Glabrolide | 90479675 | 25.84 | C30H44O4 | 468.67 | 469.33123 | 0.647 | 469.33154 | Flavone |
| 51 | Physcion | 10639 | 26.3 | C16H12O5 | 284.26 | 283.06009 | 3.851 | 283.06009 | anthraquinones |
| 52 | Glabrene | 480774 | 26.86 | C20H18O4 | 322.35 | 321.11213 | 4.125 | 321.11346 | Flavone |
| 53 | Sesamin | 72307 | 27.02 | C20H18O6 | 354.35 | 353.10196 | 4.206 | 353.10345 | Flavone |
| 54 | Licoricesaponin G2 | 14891565 | 27.3 | C42H62O17 | 838.93 | 837.39032 | 3.348 | 837.39313 | saponins |
| 55 | Glepidotin B | 442411 | 27.39 | C20H20O5 | 340.37 | 339.1227 | 3.184 | 339.12378 | Flavone |
| 56 | Licoflavonol | 5481964 | 27.56 | C20H18O6 | 354.35 | 355.11761 | -1.731 | 355.117 | Flavone |
| 57 | glycycoumarin | 5317756 | 27.58 | C21H20O6 | 368.38 | 367.11761 | 3.828 | 367.11902 | Flavone |
| 58 | Glycyrrhizic acid | 14982 | 27.97 | C42H62O16 | 822.93 | 821.39541 | 3.979 | 821.39868 | triterpenes |
| 59 | Gancaonin M | 14604078 | 28.71 | C21H20O5 | 352.4 | 351.08631 | 3.633 | 351.08759 | triterpenes |
| 60 | Licochalcone C | 9840805 | 28.74 | C21H22O4 | 338.4 | 339.15908 | -1.609 | 339.15854 | Flavone |
| 61 | glepidotin A | 5281619 | 29.11 | C20H18O5 | 338.4 | 339.1227 | -0.236 | 339.12262 | Flavone |
| 62 | Glabrone | 5317652 | 29.62 | C20H16O5 | 336.34 | 335.0914 | 4.476 | 335.0929 | Flavone |
| 63 | Glabrone | 5317652 | 29.86 | C20H16O5 | 336.34 | 337.10705 | -1.335 | 337.1066 | Flavone |
| 64 | Emodin | 3220 | 29.92 | C15H10O5 | 270.24 | 269.04444 | 5.055 | 269.04581 | anthraquinones |
| 65 | Gancaonin E | 480770 | 30.25 | C25H28O6 | 394.54 | 423.18021 | 3.367 | 423.18164 | Flavone |
| 66 | Glabrol | 11596309 | 30.55 | C25H28O4 | 392.49 | 391.19038 | 4.408 | 391.19211 | Flavone |
| 67 | glyasperin a | 5481963 | 31.35 | C25H26O6 | 422.47 | 423.18021 | 3.882 | 421.1662 | Flavone |
| 68 | Glycyrrhetinic acid | 10114 | 32.18 | C30H46O4 | 470.68 | 469.33123 | 2.927 | 469.33261 | triterpenes |
| 69 | Linolenic acid | 5280934 | 33.48 | C18H30O2 | 278.43 | 277.2162 | 4.882 | 277.21756 | Fatty Acids |
| 70 | Linoleic acid | 5280450 | 34.22 | C18H32O2 | 280.45 | 279.23185 | 4.99 | 279.23325 | Fatty Acids |
| 71 | Palmitic acid | 985 | 34.88 | C16H32O2 | 256.42 | 255.23185 | 4.558 | 255.23302 | Fatty Acids |
| 72 | oleic acid | 445639 | 35.28 | C18H34O2 | 282.46 | 281.2475 | 4.847 | 281.24887 | Fatty Acids |

In the 2023 study titled "TaoHe ChengQi decoction ameliorates sepsis-induced cardiac dysfunction through anti-ferroptosis via the Nrf2 pathway", researchers retrieved SMILES information for the components of THCQ from the PubChem database and conducted pharmacokinetic screening on the SwissADME platform. The screening criteria included meeting multiple drug-likeness principles and exhibiting high gastrointestinal absorption. Compounds that met these criteria were then subjected to target prediction, with those having a probability greater than 0 considered potential drug targets. This analytical approach provided a comprehensive chemical profile of THCQ, enhancing our understanding of its potential therapeutic effects [1].

Reference:

[1] Lu SM, Yang B, Tan ZB, Wang HJ, Xie JD, Xie MT, Jiang WH, Huang JZ, Li J, Zhang L, Tan YZ, Zhang JZ, Liu B, Wu WW, Zhang SW. TaoHe ChengQi decoction ameliorates sepsis-induced cardiac dysfunction through anti-ferroptosis via the Nrf2 pathway. Phytomedicine. 2024 Jul;129:155597. doi: 10.1016/j.phymed.2024.155597. Epub 2024 Apr 20. PMID: 38643713.

**Table 3 Description of drug composition and preparation method of THCQ in the included studies.**

| **No.** | **Study ID** | **Medicinal materials and dosage (g) of THCQ** | **Preparation Method** | **Quality control standards for drugs** | **Reported** |
| --- | --- | --- | --- | --- | --- |
| 1 | Bao YD 2013 | *Prunus persica* (L.) Batsch [Rosaceae; *Persicae Semen*] (15g), *Rheum palmatum* L. [Polygonaceae; *Rhei Radix et Rhizoma*] (12g), *Cinnamomum cassia* (L.) J.Presl [Lauraceae; *Cinnamomi ramulus*] (6g), *Natrii Sulfas* [Sodium sulfate] (6g), *Glycyrrhiza uralensis* Fisch. ex DC. [Fabaceae; *Glycyrrhizae Radix et Rhizoma*] (6g) | Prepared in accordance with the standards of the Chinese Pharmacopoeia and decocted in water | Quality control standards for drugs in compliance with the Chinese Pharmacopoeia | Yes |
| 2 | Chen Y 2021 | *Prunus persica* (L.) Batsch [Rosaceae; *Persicae Semen*] (10g), *Rheum palmatum* L. [Polygonaceae; *Rhei Radix et Rhizoma*] (10g), *Cinnamomum cassia* (L.) J.Presl [Lauraceae; *Cinnamomi ramulus*] (6g), *Natrii Sulfas* [Sodium sulfate] (6g), *Glycyrrhiza uralensis* Fisch. ex DC. [Fabaceae; *Glycyrrhizae Radix et Rhizoma*] (6g), *Citrus aurantium* L. [Rutaceae; *Fructus Aurantii Immaturus*] (15g), *Atractylodes macrocephala* Koidz. [Asteraceae; *Rhizoma Atractylodis Macrocephalae*] (10g) | Prepared in accordance with the standards of the Chinese Pharmacopoeia and decocted in water | Quality control standards for drugs in compliance with the Chinese Pharmacopoeia | Yes |
| 3 | Jin WM 2020 | *Prunus persica* (L.) Batsch [Rosaceae; *Persicae Semen*] (12g), *Rheum palmatum* L. [Polygonaceae; *Rhei Radix et Rhizoma*] (12g), *Cinnamomum cassia* (L.) J.Presl [Lauraceae; *Cinnamomi ramulus*] (6g), *Natrii Sulfas* [Sodium sulfate] (6g), *Glycyrrhiza uralensis* Fisch. ex DC. [Fabaceae; *Glycyrrhizae Radix et Rhizoma*] (12g) | Prepared in accordance with the standards of the Chinese Pharmacopoeia and decocted in water | Quality control standards for drugs in compliance with the Chinese Pharmacopoeia | Yes |
| 4 | Lan WC 2013 | *Prunus persica* (L.) Batsch [Rosaceae; *Persicae Semen*] (12g), *Rheum palmatum* L. [Polygonaceae; *Rhei Radix et Rhizoma*] (12g), *Cinnamomum cassia* (L.) J.Presl [Lauraceae; *Cinnamomi ramulus*] (6g), *Natrii Sulfas* [Sodium sulfate] (6g), *Glycyrrhiza uralensis* Fisch. ex DC. [Fabaceae; *Glycyrrhizae Radix et Rhizoma*] (6g) | Prepared in accordance with the standards of the Chinese Pharmacopoeia and decocted in water | Quality control standards for drugs in compliance with the Chinese Pharmacopoeia | Yes |
| 5 | Li H 2020 | *Prunus persica* (L.) Batsch [Rosaceae; *Persicae Semen*] (12g), *Rheum palmatum* L. [Polygonaceae; *Rhei Radix et Rhizoma*] (12g), *Cinnamomum cassia* (L.) J.Presl [Lauraceae; *Cinnamomi ramulus*] (6g), *Natrii Sulfas* [Sodium sulfate] (6g), *Glycyrrhiza uralensis* Fisch. ex DC. [Fabaceae; *Glycyrrhizae Radix et Rhizoma*] (12g) | Prepared in accordance with the standards of the Chinese Pharmacopoeia and decocted in water | Quality control standards for drugs in compliance with the Chinese Pharmacopoeia | Yes |
| 6 | Li L 2023 | *Prunus persica* (L.) Batsch [Rosaceae; *Persicae Semen*], *Rheum palmatum* L. [Polygonaceae; *Rhei Radix et Rhizoma*], *Cinnamomum cassia* (L.) J.Presl [Lauraceae; *Cinnamomi ramulus*], *Natrii Sulfas* [Sodium sulfate], *Glycyrrhiza uralensis* Fisch. ex DC. [Fabaceae; *Glycyrrhizae Radix et Rhizoma*], *Astragalus membranaceus* Fisch. ex Bunge. [Fabaceae; *Radix Astragali Mongolici*], *Gynostemma pentaphyllum* (Thunb.) Makino. [Cucurbitaceae; *Herba seu Radix Gynostemmatis Pentaphylli*], *Reynoutria japonica* Houtt. [Polygonaceae; *Radix Polygoni Cuspidati*], *Pheretima aspergillum*. [Lumbricidae; Animal-derived drug] | Prepared in accordance with the standards of the Chinese Pharmacopoeia and decocted in water | Quality control standards for drugs in compliance with the Chinese Pharmacopoeia | Yes |
| 7 | Shen JG 2021 | *Prunus persica* (L.) Batsch [Rosaceae; *Persicae Semen*] (10g), *Rheum palmatum* L. [Polygonaceae; *Rhei Radix et Rhizoma*] (10g), *Cinnamomum cassia* (L.) J.Presl [Lauraceae; *Cinnamomi ramulus*] (6g), *Natrii Sulfas* [Sodium sulfate] (6g), *Glycyrrhiza uralensis* Fisch. ex DC. [Fabaceae; *Glycyrrhizae Radix et Rhizoma*] (6g), *Citrus aurantium* L. [Rutaceae; *Fructus Aurantii Immaturus*] (15g), *Atractylodes macrocephala* Koidz. [Asteraceae; *Rhizoma Atractylodis Macrocephalae*] (10g) | Prepared in accordance with the standards of the Chinese Pharmacopoeia and decocted in water | Quality control standards for drugs in compliance with the Chinese Pharmacopoeia | Yes |
| 8 | Shi LF 2012 | *Prunus persica* (L.) Batsch [Rosaceae; *Persicae Semen*] (12g), *Rheum palmatum* L. [Polygonaceae; *Rhei Radix et Rhizoma*] (12g), *Cinnamomum cassia* (L.) J.Presl [Lauraceae; *Cinnamomi ramulus*] (6g), *Natrii Sulfas* [Sodium sulfate] (6g), *Glycyrrhiza uralensis* Fisch. ex DC. [Fabaceae; *Glycyrrhizae Radix et Rhizoma*] (6g) | Prepared in accordance with the standards of the Chinese Pharmacopoeia and decocted in water | Quality control standards for drugs in compliance with the Chinese Pharmacopoeia | Yes |
| 9 | Wang J 2021 | *Prunus persica* (L.) Batsch [Rosaceae; *Persicae Semen*] (10g), *Rheum palmatum* L. [Polygonaceae; *Rhei Radix et Rhizoma*] (10g), *Cinnamomum cassia* (L.) J.Presl [Lauraceae; *Cinnamomi ramulus*] (6g), *Natrii Sulfas* [Sodium sulfate] (6g), *Glycyrrhiza uralensis* Fisch. ex DC. [Fabaceae; *Glycyrrhizae Radix et Rhizoma*] (6g), *Citrus aurantium* L. [Rutaceae; *Fructus Aurantii Immaturus*] (15g), *Atractylodes macrocephala* Koidz. [Asteraceae; *Rhizoma Atractylodis Macrocephalae*] (10g) | Prepared in accordance with the standards of the Chinese Pharmacopoeia and decocted in water | Quality control standards for drugs in compliance with the Chinese Pharmacopoeia | Yes |
| 10 | Wang J 2024 | *Prunus persica* (L.) Batsch [Rosaceae; *Persicae Semen*] (10g), *Rheum palmatum* L. [Polygonaceae; *Rhei Radix et Rhizoma*] (15g), *Cinnamomum cassia* (L.) J.Presl [Lauraceae; *Cinnamomi ramulus*] (6g), *Natrii Sulfas* [Sodium sulfate] (6g), *Glycyrrhiza uralensis* Fisch. ex DC. [Fabaceae; *Glycyrrhizae Radix et Rhizoma*] (6g), *Paeonia lactiflora* Pall. [Paeoniaceae; *Radix Paeoniae Rubra*] (9g), *Paeonia × suffruticosa* Andrews. [Paeoniaceae; *Cortex Moutan Radicis*] (9g), *Angelica sinensis* (Oliv.) Diels. [Apiaceae; *Radix Angelicae Sinensis*] (9g) | Prepared in accordance with the standards of the Chinese Pharmacopoeia and decocted in water | Quality control standards for drugs in compliance with the Chinese Pharmacopoeia | Yes |
| 11 | Wu SL 2020 | *Prunus persica* (L.) Batsch [Rosaceae; *Persicae Semen*] (12g), *Rheum palmatum* L. [Polygonaceae; *Rhei Radix et Rhizoma*] (12g), *Cinnamomum cassia* (L.) J.Presl [Lauraceae; *Cinnamomi ramulus*] (6g), *Natrii Sulfas* [Sodium sulfate] (6g), *Glycyrrhiza uralensis* Fisch. ex DC. [Fabaceae; *Glycyrrhizae Radix et Rhizoma*] (12g) | Prepared in accordance with the standards of the Chinese Pharmacopoeia and decocted in water | Quality control standards for drugs in compliance with the Chinese Pharmacopoeia | Yes |
| 12 | Xie HY 2023 | *Prunus persica* (L.) Batsch [Rosaceae; *Persicae Semen*] (12g), *Rheum palmatum* L. [Polygonaceae; *Rhei Radix et Rhizoma*] (12g), *Cinnamomum cassia* (L.) J.Presl [Lauraceae; *Cinnamomi ramulus*] (6g), *Natrii Sulfas* [Sodium sulfate] (6g), *Glycyrrhiza uralensis* Fisch. ex DC. [Fabaceae; *Glycyrrhizae Radix et Rhizoma*] (12g) | Prepared in accordance with the standards of the Chinese Pharmacopoeia and decocted in water | Quality control standards for drugs in compliance with the Chinese Pharmacopoeia | Yes |
| 13 | Yang RY 2009 | *Prunus persica* (L.) Batsch [Rosaceae; *Persicae Semen*] (20g), *Rheum palmatum* L. [Polygonaceae; *Rhei Radix et Rhizoma*] (20g), *Cinnamomum cassia* (L.) J.Presl [Lauraceae; *Cinnamomi ramulus*] (10g), *Natrii Sulfas* [Sodium sulfate] (10g), *Glycyrrhiza uralensis* Fisch. ex DC. [Fabaceae; *Glycyrrhizae Radix et Rhizoma*] (10g) | Prepared in accordance with the standards of the Chinese Pharmacopoeia and decocted in water | Quality control standards for drugs in compliance with the Chinese Pharmacopoeia | Yes |
| 14 | Zhang X 2021 | *Prunus persica* (L.) Batsch [Rosaceae; *Persicae Semen*] (12g), *Rheum palmatum* L. [Polygonaceae; *Rhei Radix et Rhizoma*] (10g), *Cinnamomum cassia* (L.) J.Presl [Lauraceae; *Cinnamomi ramulus*] (6g), *Natrii Sulfas* [Sodium sulfate] (8g), *Glycyrrhiza uralensis* Fisch. ex DC. [Fabaceae; *Glycyrrhizae Radix et Rhizoma*] (6g), *Angelica sinensis* (Oliv.) Diels. [Apiaceae; *Radix Angelicae Sinensis*] (12), *Paeonia lactiflora* Pall. [Paeoniaceae; *Radix Paeoniae Rubra*] (15g), *Paeonia × suffruticosa* Andrews. [Paeoniaceae; *Cortex Moutan Radicis*] (15g), *Prunus armeniaca* L. var. ansu (Maxim.) Yü et Lu [Rosaceae; *Semen Armeniacae Amarum*] (10g), *Platycodon grandiflorus* (Jacq.) A.DC. [Campanulaceae; *Radix Platycodi*] (9g), *Tussilago farfara* L. [Asteraceae; *Farfarae Flos*] (10g), *Aster tataricus* L. f. [Asteraceae; *Radix Asteris Tatarici*] (10g), *Raphanus sativus* L. [Brassicaceae; *Semen Raphani Sativi*] (10g) | Prepared in accordance with the standards of the Chinese Pharmacopoeia and decocted in water | Quality control standards for drugs in compliance with the Chinese Pharmacopoeia | Yes |
| 15 | Zhu DC 2010 | *Prunus persica* (L.) Batsch [Rosaceae; *Persicae Semen*] (12g), *Rheum palmatum* L. [Polygonaceae; *Rhei Radix et Rhizoma*] (12g), *Cinnamomum cassia* (L.) J.Presl [Lauraceae; *Cinnamomi ramulus*] (6g), *Natrii Sulfas* [Sodium sulfate] (6g), *Glycyrrhiza uralensis* Fisch. ex DC. [Fabaceae; *Glycyrrhizae Radix et Rhizoma*] (6g) | Prepared in accordance with the standards of the Chinese Pharmacopoeia and decocted in water | Quality control standards for drugs in compliance with the Chinese Pharmacopoeia | Yes |
| 16 | Zhu DC 2019 | *Prunus persica* (L.) Batsch [Rosaceae; *Persicae Semen*] (10g), *Rheum palmatum* L. [Polygonaceae; *Rhei Radix et Rhizoma*] (10g), *Cinnamomum cassia* (L.) J.Presl [Lauraceae; *Cinnamomi ramulus*] (6g), *Natrii Sulfas* [Sodium sulfate] (6g), *Glycyrrhiza uralensis* Fisch. ex DC. [Fabaceae; *Glycyrrhizae Radix et Rhizoma*] (6g) | Prepared in accordance with the standards of the Chinese Pharmacopoeia and decocted in water | Quality control standards for drugs in compliance with the Chinese Pharmacopoeia | Yes |

Annotation: All drugs mentioned above were prescribed by clinical physicians in public hospitals and prepared in the pharmacy.
