# Supplementary material for: Classical formula Taohe Chengqi decoction as an adjuvant therapy for sepsis - a systematic review and meta-analysis of randomized controlled trials
Source: Front Pharmacol. 2025 Sep 2;16:1499280. doi: 10.3389/fphar.2025.1499280 (PMC12436689; doi:10.3389/fphar.2025.1499280)
Supplement: Supplementary file 2 [file Supplementaryfile5.docx]

**Figure 1 Sensitivity analysis of APACHE-Ⅱ**

**Figure 2 Sensitivity analysis of SOFA**

**Figure 3 Sensitivity analysis of WBC**

**Figure 4 Sensitivity analysis of PCT**

**Figure 5 Sensitivity analysis of CRP**

**Figure 6 Sensitivity analysis of IL-6**

**Figure 7 Sensitivity analysis of PLT**

**Figure 8 Publication bias assessment of APACHE-Ⅱ**

**Figure 9 Publication bias assessment of WBC**

**Figure 10 Publication bias assessment of PCT**
