# Supplementary material for: Classical formula Taohe Chengqi decoction as an adjuvant therapy for sepsis - a systematic review and meta-analysis of randomized controlled trials
Source: Front Pharmacol. 2025 Sep 2;16:1499280. doi: 10.3389/fphar.2025.1499280 (PMC12436689; doi:10.3389/fphar.2025.1499280)
Supplement: Supplementary file 3 [file Supplementaryfile3.docx]

**Table 1 Search strategy for the 8 databases.**

| Database | Search strategies |
| --- | --- |
| CNKI | ((SU=(脓毒症) OR SU=(脓毒血症) OR SU=(脓血症) OR SU=(败血病) OR SU=(败血症)) OR (TKA=(脓毒症) OR TKA=(脓毒血症) OR TKA=(脓血症) OR TKA=(败血病) OR TKA=(败血症))) AND ((SU=(桃仁承气) OR SU=(桃核承气)) OR (TKA=(桃仁承气) OR TKA=(桃核承气))) |
| Wanfang | ((主题:(脓毒症 or 脓毒血症 or 脓血症 or 败血病 or 败血症)) or (题名或关键词:(脓毒症 or 脓毒血症 or 脓血症 or 败血病 or 败血症)) or (摘要:(脓毒症 or 脓毒血症 or 脓血症 or 败血病 or 败血症))) and ((主题:(桃仁承气 or 桃核承气)) or (题名或关键词:(桃仁承气 or 桃核承气)) or (摘要:(桃仁承气 or 桃核承气))) |
| VIP | (M=(脓毒症 OR 脓毒血症 OR 脓血症 OR 败血病 OR 败血症) OR R=(脓毒症 OR 脓毒血症 OR 脓血症 OR 败血病 OR 败血症)) AND (M=(桃仁承气汤 OR 桃核承气汤) OR R=(桃仁承气汤 OR 桃核承气汤)) |
| SinoMed | ("脓毒症"[常用字段:智能] OR "脓毒血症"[常用字段:智能] OR "脓血症"[常用字段:智能] OR "败血病"[常用字段:智能] OR "败血症"[常用字段:智能]) AND ("桃仁承气"[常用字段:智能] OR "桃核承气"[常用字段:智能]) |
| Pubmed | #1 "Systemic Inflammatory Response Syndrome"[MeSH Terms] OR "Sepsis"[MeSH Terms] OR "Neonatal Sepsis"[MeSH Terms] OR "Sepsis-Associated Encephalopathy"[MeSH Terms] OR "Systemic Inflammatory Response Syndrome"[Title/Abstract] OR "Sepsis"[Title/Abstract] OR "Neonatal Sepsis"[Title/Abstract] OR "Sepsis-Associated Encephalopathy"[Title/Abstract] OR "Inflammatory Response Syndrome, Systemic"[Title/Abstract] OR "Bloodstream Infection  "[Title/Abstract] OR "Infection, Bloodstream"[Title/Abstract] OR "Septicemia"[Title/Abstract] OR "Blood Poisoning"[Title/Abstract] OR "Poisoning, Blood"[Title/Abstract] OR "Severe Sepsis"[Title/Abstract] OR "Sepsis, Severe"[Title/Abstract] OR "Pyemia"[Title/Abstract] OR "Pyaemia"[Title/Abstract] OR "Pyohemia"[Title/Abstract] OR "Sepses"[Title/Abstract]  #2 "taorenchengqi"[Title/Abstract] OR "taoren chengqi"[Title/Abstract] OR "tao ren cheng qi"[Title/Abstract] OR "taohechengqi"[Title/Abstract] OR "taohe chengqi"[Title/Abstract] OR "tao he cheng qi"[Title/Abstract] OR "THCQ"[Title/Abstract] OR "TRCQ"[Title/Abstract]  #3 #1 AND #2 |
| Cochrane Library | #1 MeSH descriptor: [Systemic Inflammatory Response Syndrome] explode all trees  #2 MeSH descriptor: [Sepsis] explode all trees  #3 MeSH descriptor: [Neonatal Sepsis] explode all trees  #4 MeSH descriptor: [Sepsis-Associated Encephalopathy] explode all trees  #5 (Systemic Inflammatory Response Syndrome):ti,ab,kw  #6 (Sepsis):ti,ab,kw  #7 (Neonatal Sepsis):ti,ab,kw  #8 (Sepsis-Associated Encephalopathy):ti,ab,kw  #9 (Inflammatory Response Syndrome, Systemic):ti,ab,kw  #10 (Bloodstream Infection):ti,ab,kw  #11 (Infection, Bloodstream):ti,ab,kw  #12 (Septicemia):ti,ab,kw  #13 (Blood Poisoning):ti,ab,kw  #14 (Poisoning, Blood):ti,ab,kw  #15 (Severe Sepsis):ti,ab,kw  #16 (Sepsis, Severe):ti,ab,kw  #17 (Pyemia):ti,ab,kw  #18 (Pyaemia):ti,ab,kw  #19 (Pyohemia):ti,ab,kw  #20 (Sepses):ti,ab,kw  #21 #1 OR #2 OR #3 OR #4 OR #5 OR #6 OR #7 OR #8 OR #9 OR #10 OR #11 OR #12 OR #13 OR #14 OR #15 OR #16 OR #17 OR #18 OR #19 OR #20  #22 (taorenchengqi):ti,ab,kw  #23 (taoren chengqi):ti,ab,kw  #24 (tao ren cheng qi):ti,ab,kw  #25 (taohechengqi):ti,ab,kw  #26 (taohe chengqi):ti,ab,kw  #27 (tao he cheng qi):ti,ab,kw  #28 (THCQ):ti,ab,kw  #29 (TRCQ):ti,ab,kw  #30 #22 OR #23 OR #24 OR #25 OR #26 OR #27 OR #28 OR #29  #31 #21 AND #30 |
| Embase | #1 'systemic inflammatory response syndrome'/exp OR 'systemic inflammatory response syndrome' OR 'sepsis'/exp OR 'sepsis' OR 'neonatal sepsis'/exp OR 'neonatal sepsis' OR 'sepsis-associated encephalopathy'/exp OR 'sepsis-associated encephalopathy' OR 'inflammatory response syndrome, systemic' OR 'bloodstream infection'/exp OR 'bloodstream infection' OR 'infection, bloodstream' OR 'septicemia'/exp OR 'septicemia' OR 'blood poisoning' OR 'poisoning, blood' OR 'severe sepsis'/exp OR 'severe sepsis' OR 'sepsis, severe' OR 'pyemia'/exp OR 'pyemia' OR 'pyaemia'/exp OR 'pyaemia' OR 'pyohemia'/exp OR 'pyohemia' OR 'sepses'  #2 'taorenchengqi' OR 'taoren chengqi' OR 'tao ren cheng qi' OR 'taohechengqi' OR 'taohe chengqi' OR 'tao he cheng qi' OR 'thcq' OR 'trcq'  #3 #1 AND #2 |
| Web of Science | #1 (((((((ALL=(taorenchengqi)) OR ALL=(taoren chengqi)) OR ALL=(tao ren cheng qi)) OR ALL=(taohechengqi)) OR ALL=(taohe chengqi)) OR ALL=(tao he cheng qi)) OR ALL=(THCQ)) OR ALL=(TRCQ)  #2 (((((((((((((((ALL=(Systemic Inflammatory Response Syndrome)) OR ALL=(Sepsis)) OR ALL=(Neonatal Sepsis)) OR ALL=(Sepsis-Associated Encephalopathy)) OR ALL=(Inflammatory Response Syndrome, Systemic)) OR ALL=(Bloodstream Infection)) OR ALL=(Infection, Bloodstream)) OR ALL=(Septicemia)) OR ALL=(Blood Poisoning)) OR ALL=(Poisoning, Blood)) OR ALL=(Severe Sepsis)) OR ALL=(Sepsis, Severe)) OR ALL=(Pyemia)) OR ALL=(Pyaemia)) OR ALL=(Pyohemia)) OR ALL=(Sepses)  #3 #1 AND #2 |
