# Supplementary material for: Classical formula Taohe Chengqi decoction as an adjuvant therapy for sepsis - a systematic review and meta-analysis of randomized controlled trials
Source: Front Pharmacol. 2025 Sep 2;16:1499280. doi: 10.3389/fphar.2025.1499280 (PMC12436689; doi:10.3389/fphar.2025.1499280)
Supplement: Supplementary file 4 [file Supplementaryfile2.docx]

**Checklist of Items for Reporting Systematic Reviews of Chinese Herbal Medicines (PRISMA-CHM)**

| Section/Topic | Item Number | PRISMA Checklist Item | Extension for CHM | Corresponding section |
| --- | --- | --- | --- | --- |
| TITLE | | | | |
| Title | 1 | Identify the report as a systematic review, meta-analysis, or both. | 1a. Specify the name of the CHM intervention, in terms of (1) Chinese medicinal substance(s), and/or (2) CHM formula(s). 1b. State whether the review targets (1) Western medicine-defined disease(s), or (2) Western medicine-defined disease(s) with specific TCM Pattern(s), or (3) TCM Pattern(s). | Title |
| ABSTRACT | | | | |
| Structured summary | 2 | Provide a structured summary including, as applicable: background; objectives; data sources; study eligibility criteria, partici- pants, and interventions; study appraisal and synthesis methods; results; limita- tions; conclusions and implications of key findings; systematic review registration number. | Provide the name and form of the CHM intervention(s) used, and the TCM Pattern applied (if any). | Abstract |
| INTRODUCTION | | | | |
| Rationale | 3 | Describe the rationale for the review in the  context of what is already known. | 3a. State the rationale of using particular CHM intervention(s) to target the specific disease(s) and/or TCM Pattern (if any),  ideally in terms of TCM theory.  3b. State the importance of the review. | Introduction |
| Objectives | 4 | Provide an explicit statement of questions being addressed with reference to participants, interventions, comparisons, outcomes, and study design (PICOS). | State whether the CHM intervention(s) targets a Western medicine-defined disease, a TCM Pattern, or a Western medicine-defined disease with a specific TCM Pattern. | Introduction |
| METHODS | | | | |
| Protocol and registration | 5 | Indicate if a review protocol exists, if and where it can be accessed (e.g., Web address), and, if available, provide registration information including registration number. |  | Materials and methods |
| Eligibility criteria | 6 | Specify study characteristics (e.g., PICOS, length of follow-up) and report characteristics (e.g., years considered, language, publication status) used as criteria for eligibility, giving rationale. | 6a. As applicable, state whether participants with a specific TCM Pattern will be included, in terms of (1) diagnostic criteria, and (2) inclusion and exclusion criteria. All criteria utilized should be universally recognized, or reference(s) where detailed explanation(s) can be found should be given.  6b. Specify the detailed requirements of CHM intervention(s), considering (1) types, such as whether CHM formulas are fixed, individualized, or patent proprietary; (2) composition, such as main herb(s) in a CHM formula; (3) dosage form, such as decoction, granules, powder; and (4) treatment duration.  6c. Specify the types of control group(s), if any, such as placebo control, active control, other treatment control or blank control.  6d. State whether TCM-related outcome(s) will be included, and if so, describe the change of degree and scope of symptoms and signs related to TCM Pattern differentiation. | Materials and methods: Eligibility criteria |
| Information sources | 7 | Describe all information sources (e.g., data- bases with dates of coverage, contact with study authors to identify additional studies) in the search and date last sear- ched. |  | Materials and methods: Search strategy |
| Search | 8 | State full electronic search strategy for at least one database, including any limits used, such that it could be repeated. |  | Materials and methods: Search strategy |
| Study selection | 9 | State the process for selecting studies (i.e., screening, eligibility, included in systematic review, and, if applicable, included in the meta-analysis). |  | Materials and methods: Research selection |
| Data collection process | 10 | Describe method of data extraction from reports (e.g., piloted forms, indepen- dently, in duplicate) and any processes for obtaining and confirming data from investigators. |  | Materials and methods: Research selection |
| Data items | 11 | List and define all variables for which data were sought (e.g., PICOS, funding sources) and any assumptions and simplifi-cations made. | 11a. State details of the participants with a specific TCM Pattern (if any), considering (1) diagnostic criteria; and (2)  baseline characteristics.  11b. State details of the CHM intervention(s), including (1) name, source, and dosage form; (2) name, source, processing  method, and dosage of each medical substance, if applicable, names of the parts of the substances; (3) quality control information; (4) dosage, administration route and time; (5) information about the production method, authentication method, and safety assessment, if any; (6) for CHM formulas, the principles, rationale, and interpretation of forming and/or modifying the formula; and (7) for patented proprietary CHM formulas, the name of the product and manufacturer.  11c. State details of any placebo of CHM used, considering (1) if/how it is physically identical and pharmacologically inert; (2) administration route, regimen, and dosage; (3) success of blinding, if any.  11d. State the TCM-related outcome (if any), considering (1) name and measuring methods; (2) measuring time points and length of follow-up, if applicable. | Materials and methods: Eligibility criteria |
| Risk of bias in individual studies | 12 | Describe methods used for assessing risk of bias of individual studies (including specification of whether this was done at the study or outcome level), and how this information is to be used in any data synthesis. |  | Materials and methods: Quality assessment |
| Summary measures | 13 | State the principal summary measures (e.g., risk ratio, difference in means). |  | Materials and methods: Strategy of data synthesis |
| Synthesis of results | 14 | Describe the methods of handling data and combining results of studies, if done, in- cluding measures of consistency (e.g., I2) for each meta-analysis. | When combining trial results from different studies, describe whether CHM intervention(s) matched the TCM Pattern(s) of participants, if applicable. | Materials and methods: Strategy of data synthesis |
| Risk of bias across studies | 15 | Specify any assessment of risk of bias that may affect the cumulative evidence (e.g., publication bias, selective reporting within studies). |  | Materials and methods: Strategy of data synthesis |
| Additional analyses | 16 | Describe methods of additional analyses (e.g., sensitivity or subgroup analyses, meta-regression), if done, indicating which were pre-specified. | Describe methods of subgroup analyses in terms of the CHM intervention(s) and participants, considering at least (1) the types, composi- tions, dosage, dosage form, and treatment duration of the CHM intervention(s); and (2) participants with different TCM Patterns, if any | Materials and methods: Strategy of data synthesis |
| RESULTS | | | | |
| Study selection | 17 | Give numbers of studies screened, assessed for eligibility, and included in the review, with reasons for exclusions at each stage, ideally with a flow diagram. |  | Results: Database search |
| Study characteristics | 18 | For each study, state characteristics for which data were extracted (e.g., study size, PICOS, follow-up period) and provide the citations. | 18a. State characteristics of participants with a specific TCM Pattern (if any), including diagnostic criteria and baseline data.  18b. State characteristics of the CHM intervention(s), including (1) name, source, and dosage form; (2) name, source, processing method, and dosage of each medical substance; (3) quality control information; (4) dosage, administration route and time; (5) information about the production method, authentication method, and safety assessment, if available; (6) for CHM formulas, how the formula has been modified, if applicable; and (7) for patent proprietary CHM formulas, the name of the product and manufacturer.  18c. State characteristics of the placebo of CHM (if any), including (1) whether physically identical and pharmacologically inert; (2) administration route, regimen, and dosage; and (3) success of blinding.  18d. State characteristics of the TCM-related outcome (if any), including (1) name and measuring methods; (2) measuring time points and length of follow-up | Results: Study characteristics |
| Risk of bias within studies | 19 | Present data on risk of bias of each study and, if available, any outcome-level assessment (see Item 12). |  | Results: ROB 2.0 analysis |
| Results of individual studies | 20 | For all outcomes considered (benefits or harms), present, for each study: (a) simple summary data for each intervention group and (b) effect estimates and confidence intervals, ideally with a forest plot. |  | Supplementary Appendix S4 |
| Synthesis of results | 21 | Present results of each meta-analysis done, including confidence intervals and mea- sures of consistency | Give results of each meta-analysis based on the consistency of PICOS, considering (1) par- ticipants with TCM Patterns, if any; (2) CHM intervention(s); (3) comparators (e.g. CHM placebo); and (4) TCM-related outcome(s), if any | Results: Table 3 |
| Risk of bias across studies | 22 | Present results of any assessment of risk of bias across studies (see Item 15). |  | Results: ROB 2.0 analysis |
| Additional analysis | 23 | Give results of additional analyses, if done (e.g., sensitivity or subgroup analyses, meta-regression [see Item 16]). | Give results of subgroup analyses based on the different categories of CHM intervention(s) and participants with TCM Patterns (if any), if done. | Results: Sensitivity analysis; Publication bias assessment |
| DISCUSSION | | | | |
| Summary of evidence | 24 | Summarize the main findings including the strength of evidence for each main outcome; consider their relevance to key groups (e.g., health care providers, users, and policy makers). | Summarize how the CHM intervention(s) worked on different TCM Pattern(s) or Western medicine–defined disease(s) with  specific TCM Pattern(s). Interpret the main findings in terms of TCM theory, if applicable. | Discussion: Findings from systematic review |
| Limitations | 25 | Discuss limitations at study and outcome level (e.g., risk of bias), and at review level (e.g., incomplete retrieval of identified research, reporting bias). |  | Discussion: Strengths and limitations |
| Conclusions | 26 | Provide a general interpretation of the results in the context of other evidence, and implications for future research. | When the review targets TCM Pattern(s), or Western medicine-defined disease(s) with specific TCM Pattern(s), a general interpre- tation of the results about the relationship of the CHM intervention(s) and TCM Pattern(s) should be provided. | Discussion: Implications for research |
| FUNDING | | | | |
| Funding | 27 | Describe sources of funding for the system- atic review and other support (e.g., sup- ply of data); role of funders for the systematic review. |  | Funding |
