# Supplementary material for: Classical formula Taohe Chengqi decoction as an adjuvant therapy for sepsis - a systematic review and meta-analysis of randomized controlled trials
Source: Front Pharmacol. 2025 Sep 2;16:1499280. doi: 10.3389/fphar.2025.1499280 (PMC12436689; doi:10.3389/fphar.2025.1499280)
Supplement: Supplementary file 5 [file Supplementaryfile4.docx]

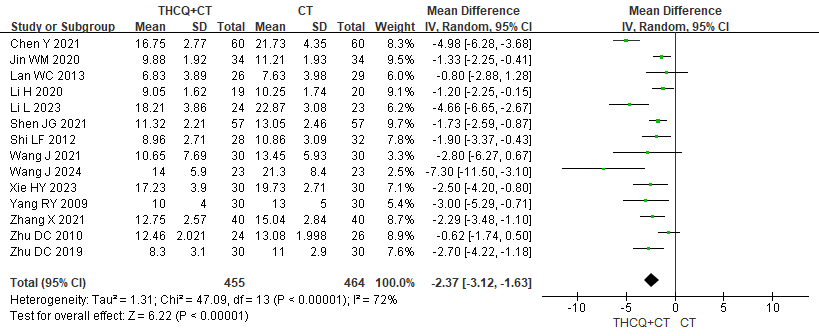


**(A): APACHE-Ⅱ**


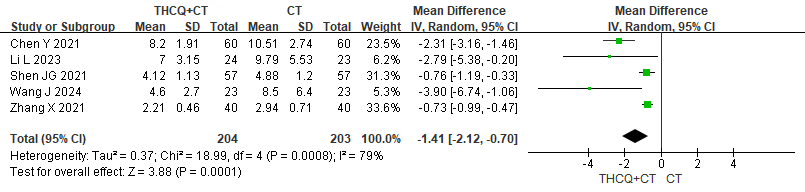


**(B): SOFA**

**Figure 1 Forest plots of the comparison on disease severity outcomes. (A): APACHE-Ⅱ, (B): SOFA.**


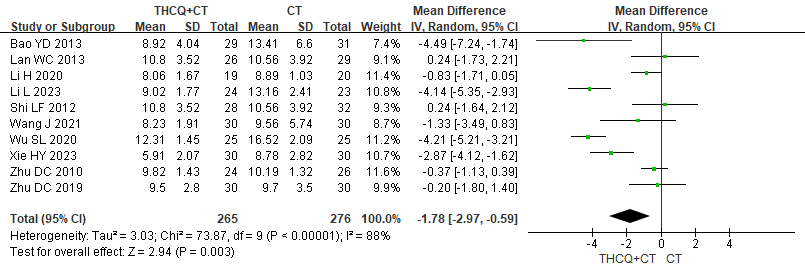


**(A): WBC**


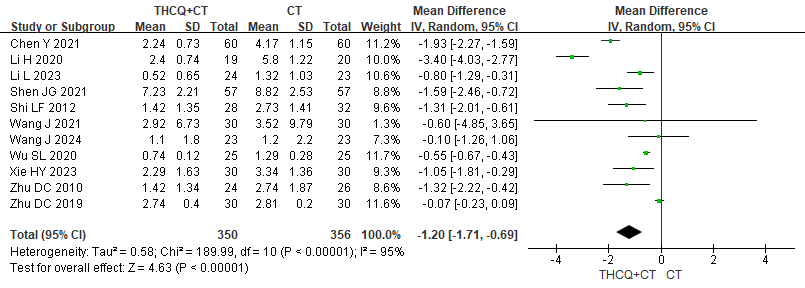


**(B): PCT**


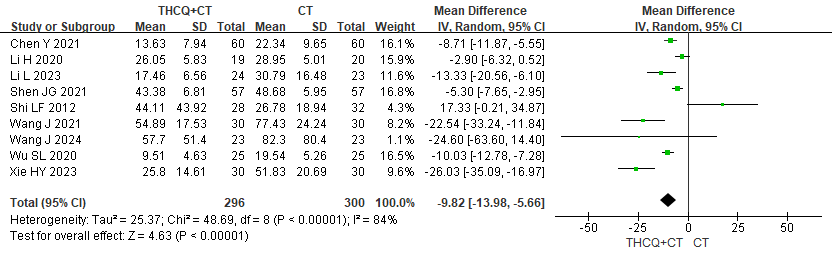


**(C): CRP**


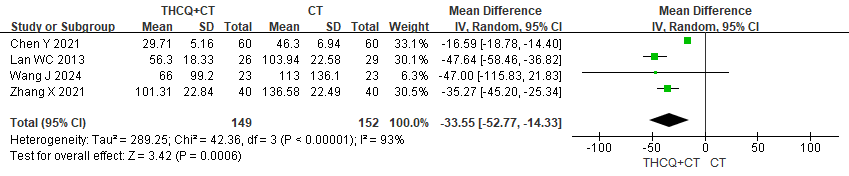


**(D): IL-6**


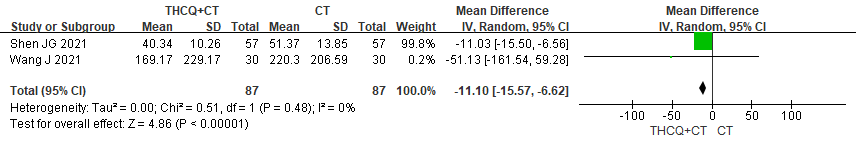


**(E): SAA**


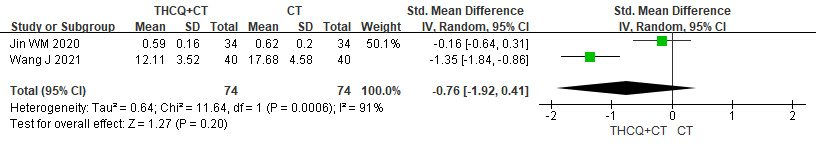


**(F): Endotoxin**

**Figure 2 Forest plots of the comparison on inflammation outcomes. (A): WBC, (B): PCT, (C): CRP, (D): IL-6, (E): SAA, (F): Endotoxin.**


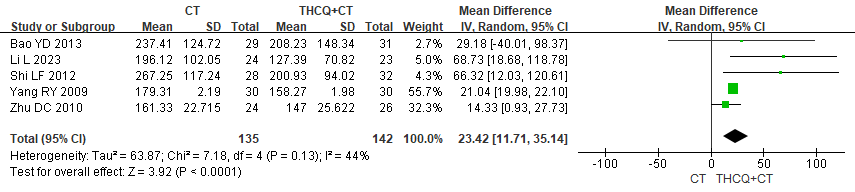


1. **: PLT**


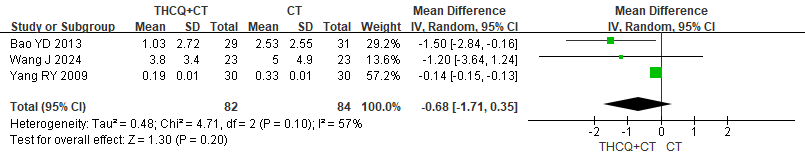


**(B): D-II**

**Figure 3 Forest plots of the comparison on coagulation outcomes. (A): PLT, (B): D-II.**
